# Unsupervised machine learning identifies distinct SLE patient endotypes with differential response to belimumab

**DOI:** 10.1093/rheumatology/keaf215

**Published:** 2025-04-17

**Authors:** Roberto Depascale, Raffaele Da Mutten, Julius Lindblom, Nursen Cetrez, Leonardo Palazzo, Luca Iaccarino, Andrea Doria, Dionysis Nikolopoulos, Mariele Gatto, Ioannis Parodis

**Affiliations:** Division of Rheumatology, Department of Medicine DIMED, University of Padua, Padua, Italy; Division of Rheumatology, Department of Medicine Solna, Karolinska Institutet, Karolinska University Hospital, Center for Molecular Medicine (CMM), Stockholm, Sweden; Division of Rheumatology, Department of Medicine Solna, Karolinska Institutet, Karolinska University Hospital, Center for Molecular Medicine (CMM), Stockholm, Sweden; Division of Rheumatology, Department of Medicine Solna, Karolinska Institutet, Karolinska University Hospital, Center for Molecular Medicine (CMM), Stockholm, Sweden; Division of Rheumatology, Department of Medicine Solna, Karolinska Institutet, Karolinska University Hospital, Center for Molecular Medicine (CMM), Stockholm, Sweden; Division of Rheumatology, Department of Medicine DIMED, University of Padua, Padua, Italy; Division of Rheumatology, Department of Medicine DIMED, University of Padua, Padua, Italy; Division of Rheumatology, Department of Medicine Solna, Karolinska Institutet, Karolinska University Hospital, Center for Molecular Medicine (CMM), Stockholm, Sweden; Academic Rheumatology Centre, Department of Clinical and Biological Sciences, University of Turin, AO Mauriziano di Torino, Turin, Italy; Division of Rheumatology, Department of Medicine Solna, Karolinska Institutet, Karolinska University Hospital, Center for Molecular Medicine (CMM), Stockholm, Sweden; Department of Rheumatology, Faculty of Medicine and Health, Örebro University, Örebro, Sweden

**Keywords:** SLE, machine learning, B cells, belimumab

## Abstract

**Objective:**

To determine SLE endotypes according to B cell immunophenotyping and serological profile and assess endotypes’ response to belimumab.

**Methods:**

We analysed data from 796 patients with SLE from the phase III trial BLISS-SC. Unsupervised machine learning employing factor analysis of mixed data (FAMD) and subsequent clustering determined endotypes based on B cell immunophenotyping and serological profiles. Cox regression was used to assess belimumab efficacy on inducing lupus low disease activity state (LLDAS) and definitions of remission in SLE (DORIS) remission within clusters.

**Results:**

Three endotypes were determined. Compared with each other, cluster 1 (*n* = 191) displayed higher proportions of CD19^+^CD24b^+^CD27^+^ regulatory B cells (mean ± SD: 35.9%±12.6%), CD19^+^CD20^+^CD27^+^ bulk memory B cells (32.2%±9.9%), CD19^+^CD20^+^CD69^+^ activated B cells (0.2%±0.3%), CD19^+^CD20^−^CD138^+^ long-lived plasma cells (0.7%±1.0%) and CD19^+^CD38b^+^CD27b^+^ SLE-associated plasma cells (6.6%±7.0%). Cluster 2 (*n* = 366) displayed higher proportions of CD19^+^CD24b^bright^CD38b^bright^CD27^−^ transitional B cells (6.3%±9.0%) and CD19^+^CD20^+^CD27^−^ naïve B cells (85.4%±7.2%), and lower proportions of CD19^+^CD20^−^CD138^+^ peripheral long-lived plasma cells (0.2%±0.3%) and CD19^+^CD38b^+^CD27b^+^ SLE-associated plasma cells (1.6%±2.0%). Cluster 3 (*n* = 239) displayed a higher proportion of CD19^+^CD20^+^CD138^+^ short-lived plasma cells (0.1%±0.1%) and higher serological activity, being enriched in anti-double stranded(ds)DNA, anti-ENAs, antiphospholipid antibodies and hypocomplementemia. Use of belimumab was superior to placebo in inducing sustained LLDAS [hazard ratio (HR): 2.22; 95% CI: 1.18–4.17; *P* = 0.014] and DORIS remission (HR: 3.45; 95% CI: 1.2–9.94; *P* = 0.022) in cluster 2.

**Conclusion:**

Three distinct SLE endotypes were identified based on B cell immunophenotyping and serological profiles, showing differential benefit from belimumab therapy.

Rheumatology key messages

- Three SLE endotypes were identified using B cell immunophenotyping and serological profiles.
- SLE endotypes exhibited unique immunophenotypic and serological features and showed differential therapeutic responses to belimumab.
- Transitional and naïve B cell enrichment marked belimumab benefit for sustained LLDAS and DORIS remission.

## Introduction

SLE is a chronic autoimmune disease characterized by the involvement of multiple organ systems, a wide range of clinical symptoms, as well as varying disease course and degree of severity [1]. The SLE course typically has a relapsing-remitting pattern, with each flare potentially contributing to additional organ damage and increased morbidity, which is closely linked to mortality [2]. SLE can manifest with potentially severe clinical conditions, e.g. kidney or CNS involvement, or milder features such as skin or joint disease.

The therapeutic armamentarium for SLE primarily includes glucocorticoids, antimalarial agents and a variety of immunosuppressants that hamper inflammation [3]. More modern targeted therapies include those directed against B cells [4] or the type I IFN receptor [5]. Belimumab, a humanized monoclonal antibody binding to the soluble form of B cell activating factor (BAFF), was the first approved biological agent for SLE patients [6]. Its proven efficacy in multiple phase III trials [7–10] underscored the central role of B cells in SLE pathogenesis [11].

B cells contribute to SLE pathogenesis not only as autoantibody producers but also through their pivotal role in cytokine production and antigen presentation [12]. Belimumab primarily impacts B cells in early maturation stages. Previous studies have highlighted that inhibiting BAFF leads to specific alterations in circulating B cell and plasma cell subsets [13], linked to patterns of clinical response [12, 14, 15]. Loss of tolerance and production of SLE-associated autoantibodies against nuclear antigens are central to SLE pathogenesis [16]. Prior research has endeavoured to stratify SLE patients into distinct endotypes based on their unique serological profiles [17–19], but how B cell subtypes could contribute to such patient characterization remains unclear.

A more refined characterization of SLE patient subgroups might enhance our understanding of underlying pathogenesis and serve as a guide in therapeutic decisions. We employed unsupervised machine learning (ML) with the aim of identifying clusters of SLE patients based on peripheral B cell and plasma cell subsets along with traditional serological markers and linking them to clinical phenotypes and patterns of response to belimumab therapy.

## Materials and methods

### Patient population

We analysed longitudinal data from patients with active SLE who participated in the randomized controlled trial (RCT) BLISS-SC (NCT01484496) [9] comparing subcutaneous belimumab with placebo as add-on to non-biological standard therapy (ST), including antimalarial agents, glucocorticoids, immunosuppressive agents or combinations thereof. The study population (*n* = 796) was selected to have complete records on B cell subset counts, serological markers and data for the determination of clinical phenotypes and response to treatment. For recruitment in BLISS-SC, all study participants were required to have a Safety of Estrogens in Lupus Erythematosus National Assessment-SLEDAI (SELENA-SLEDAI) score ≥8 [20] and had to be autoantibody positive (antinuclear antibody titre ≥1:80 and/or anti-dsDNA antibody level ≥30 IU/ml at screening). All patients were required to have been on stable doses of ST for at least 30 days prior to treatment initiation. The trial intervention comprised belimumab 200 mg or placebo administered subcutaneously weekly on top of ST through week 52.

### B cell subsets and serological markers

Peripheral B cell subsets at baseline were determined by ﬂow cytometry [9] and were classified as per study protocol into total peripheral CD19^+^CD20^+^ B cells, CD19^+^CD20^+^CD69^+^ activated B cells, CD19^+^CD20^+^CD27^−^ naïve B cells, CD19^+^CD20^+^CD27^+^ bulk memory B cells, CD19^+^CD27^+^CD24^bright^ regulatory B cells, CD19^+^CD27^−^CD24^bright^CD38^bright^ transitional B cells, CD19^+^CD20^+^CD138^+^ short-lived plasma cells, CD19^+^CD20^−^CD138^+^ long-lived plasma cells and CD19^+^CD38^bright^CD27^bright^ SLE-associated plasma cells [21, 22]. Autoantibody profiles were determined based on presence or absence of anti-dsDNA (levels >30 IU/ml), anti-Sm (levels >15 IU/ml), anticardiolipin IgM (levels >12 IU/ml) and anticardiolipin IgG antibodies (levels >14 IU/ml), and low *vs* normal/high complement C3 or C4 levels (reference values for C3: 90–180 mg/dl; reference values for C4: 10–40 mg/dl) [23].

### Evaluation of response to treatment and outcome measures

We analysed attainment of lupus low disease activity state (LLDAS) [24, 25] and remission according to the DORIS definition [26, 27]. LLDAS was defined as a SLEDAI 2000 (SLEDAI-2K) ≤4, excluding major organ activity or fever, no new activity in any descriptor since the previous assessment, SELENA-SLEDAI physician global assessment (PGA) ≤1 (scale: 0–3), a glucocorticoid dose ≤7.5 mg/day of prednisone equivalents, and standard doses of immunosuppressive drugs or approved biological agents [24].

DORIS remission was defined as clinical SLEDAI-2K = 0 and SELENA-SLEDAI PGA <0.5 (scale: 0–3), while allowing serological activity and a glucocorticoid dose ≤5 mg/day of prednisone equivalents, and standard doses of immunosuppressive drugs or approved biological agents [27].

Sustained LLDAS or DORIS remission was defined as LLDAS or DORIS remission at two or more consecutive visits, which in BLISS-SC were scheduled every fourth week, that was maintained through week 52. We also calculated mean prednisone dose from baseline through week 52 for use in the models.

Organ damage was estimated using the SLICC/ACR Damage Index (SDI) [28].

### Ethics

Data from the BLISS trials were made available by GlaxoSmithKline (Uxbridge, UK) through the Clinical Study Data Request (CSDR) consortium. The trial protocols were approved by regional ethics review boards at all recruiting centres and complied with the ethical principles of the Declaration of Helsinki. Written informed consent was obtained from all study participants prior to enrolment. The present research was approved by the Swedish Ethical Review Authority (registration number: 2019–05498).

### Statistical analysis

Descriptive statistics are reported as numbers (percentage) or mean (SD), while medians (interquartile range) are indicated in case of non-normal distributions. *P* values <0.05 were deemed statistically significant.

Factor analysis of mixed data (FAMD) was employed to reduce dimensionality to handle both categorical and continuous variables. Subsequently, the elbow method [29] was applied to identify the optimal number of clusters, resulting in three. After FAMD, the first five dimensions were used as input for k-means clustering. Quantile-based initialization of the cluster centres was used. Mean Silhouette score of each datapoint was reported.

To assess the robustness of the clustering approach, we additionally split the dataset into training (70%) and testing (30%) subsets. FAMD was performed on the training data, and the transformation was applied to the testing data. K-means clustering was then conducted on the transformed data, and the relative distribution of B cell levels across clusters was compared (Supplementary Fig. S1, available at *Rheumatology* online; Supplementary Table S1, available at *Rheumatology* online). Given the modest sample size and the goal of capturing complex differences across relatively small clusters, the full dataset was used for the final clustering. As described above, the number of clusters was determined using the elbow method, and the mean Silhouette score was reported to mitigate potential overfitting.

Clusters were visualized using t-distributed stochastic neighbour embedding (t-SNE) plots. Multigroup comparisons across clusters were conducted for demographics, routine clinical features, medications, SLEDAI-2K scores and SDI scores at baseline. Post-hoc analysis was performed to identify differences between pairs of clusters upon significant differences across the three clusters. We utilized one-way analysis of variance (ANOVA) for continuous variables and Pearson’s chi-squared test (*χ*^2^) for categorical variables in both across-cluster and pairwise comparisons.

Longitudinal comparisons in relation to LLDAS and DORIS remission were conducted at 4-week intervals from baseline through week 52, adjusting for age, sex, ancestry and belimumab treatment.

Finally, proportional hazards (Cox) regression analysis was used to determine (i) the probability of attaining sustained LLDAS or sustained DORIS remission across clusters and (ii) the benefit conferred from belimumab in attaining these outcomes within each one of the three clusters (separate models). All models were multivariable with a priori selected covariates, i.e. age, sex, ancestry and belimumab use. The former models also included each one of the clusters with the other two as the reference comparator. All analyses were performed using the R Statistical Software version 4.2.3 (R Foundation for Statistical Computing, Vienna, Austria).

## Results

### Unsupervised analysis reveals three endotypes based on B cell subsets and serological profiles

**Figure 1.**
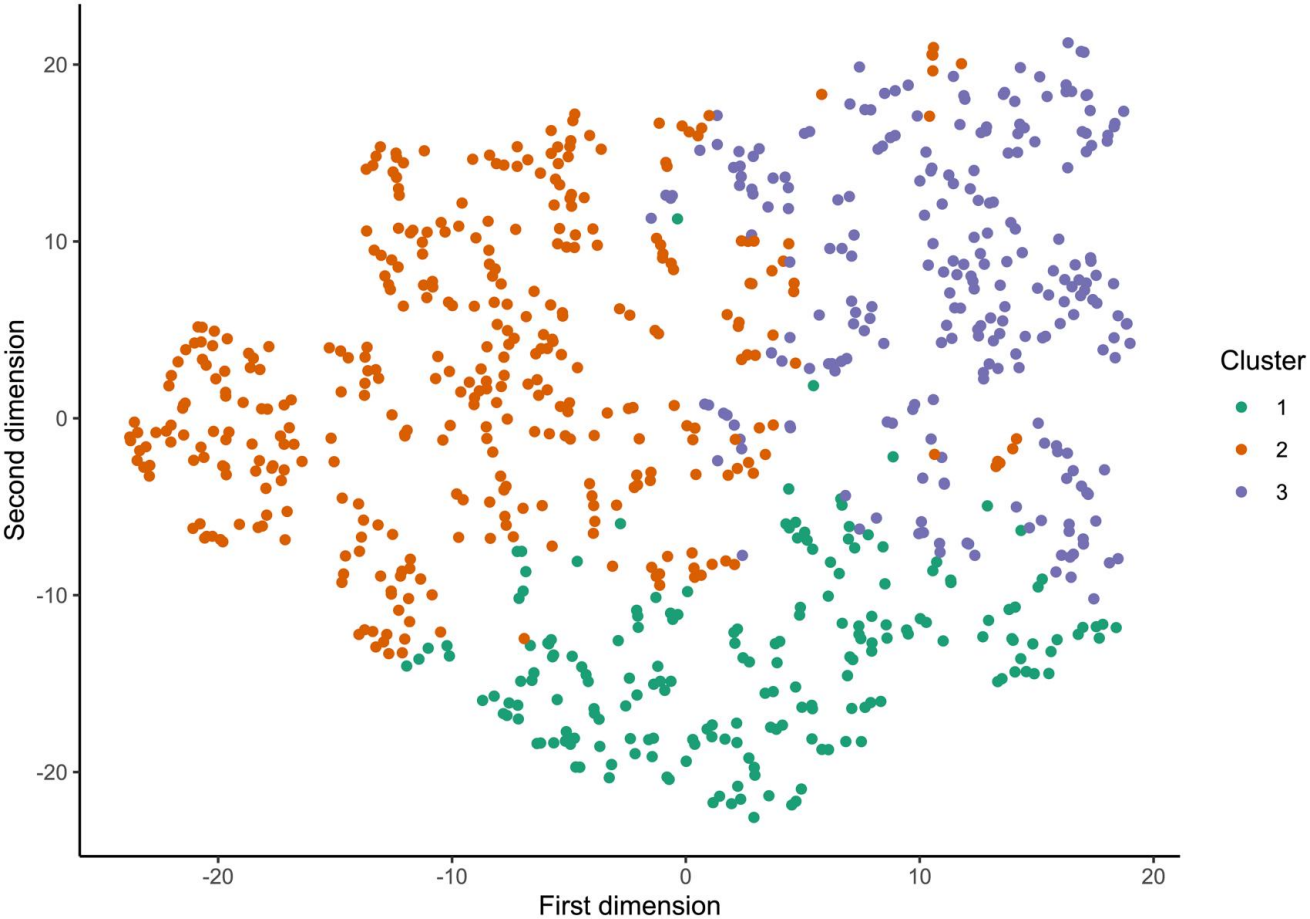
t-SNE plot of patient clusters. T-distributed stochastic neighbour embedding (t-SNE) plot depicting the three clusters of patients with SLE identified by an unsupervised analysis using B cell subsets and serology

We performed unsupervised ML analysis to stratify SLE patients into distinct groups based on B cell immunophenotyping and autoantibody profile. Three distinct clusters were determined (Fig. 1). The demographic, clinical, B cell subset and autoantibody-based characterization of these clusters is detailed in Table 1. Silhouette score (mean % ± SD %) across all datapoints was 0.28 ± 0.14. The distribution of all items per cluster is reported in Supplementary Tables S2–S10, available at *Rheumatology* online. Supplementary Tables S11–S19, available at *Rheumatology* online, show across-clusters and pairwise comparisons of the data.

**Table 1.** Demographics, clinical, B cell and serological data of the study population

	All patients (*N* = 796)	Cluster 1 (*N* = 191)	Cluster 2 (*N* = 366)	Cluster 3 (*N* = 239)	*P* value
Demographics					
Age; mean (SD)	38.55 (12.36)	38.86 (12.50)	41.20 (12.44)	34.26 (10.91)	**<0.001**
Female sex; *n* (%)	753 (94.6)	181 (94.8)	343 (93.7)	229 (95.8)	0.532
Ethnicity; *n* (%)					
Asian	180 (22.6)	39 (20.4)	73 (19.9)	68 (28.5)	**0.036**
Black/African American	77 (9.7)	12 (6.3)	37 (10.1)	28 (11.7)	0.155
Indigenous American	61 (7.7)	14 (7.3)	27 (7.4)	20 (8.4)	0.887
White/Caucasian	478 (60.1)	126 (66.0)	229 (62.6)	123 (51.5)	**0.004**
Medications at baseline					
Prednisone equivalent dose; mean (SD)	11.01 (8.49)	12.85 (7.78)	9.60 (8.85)	11.69 (8.14)	**<0.001**
Antimalarial agents; *n* (%)	550 (69.1)	123 (64.4)	264 (72.1)	163 (68.2)	0.162
Azathioprine; *n* (%)	155 (19.5)	64 (33.5)	50 (13.7)	41 (17.2)	**<0.001**
MTX; *n* (%)	85 (10.7)	12 (6.3)	48 (13.1)	25 (10.5)	**0.046**
Mofetil mycophenolate; *n* (%)	100 (12.6)	11 (5.8)	41 (11.2)	48 (20.1)	**<0.001**
Cyclophosphamide; *n* (%)	4 (0.5)	2 (1.0)	1 (0.3)	1 (0.4)	0.460
Tacrolimus; *n* (%)	25 (3.1)	2 (1.0)	14 (3.8)	9 (3.8)	0.163
Ciclosporin; *n* (%)	14 (1.8)	3 (1.6)	8 (2.2)	3 (1.3)	0.678
Leflunomide; *n* (%)	9 (1.1)	2 (1.0)	6 (1.6)	1 (0.4)	0.378
Belimumab 200 mg subcutaneous; *n* (%)	528 (66.3)	123 (64.4)	242 (66.1)	163 (68.2)	0.704
B cell immunophenotypes					
CD19^+^CD20^+^CD27^−^ naïve B cells (%); mean (SD)	77.44 (14.76)	56.78 (11.48)	85.39 (7.20)	81.78 (9.18)	**<0.001**
CD19^+^CD20^+^CD27^+^ bulk memory B cells (%); mean (SD)	16.45 (11.39)	32.15 (9.92)	11.41 (6.22)	11.60 (5.94)	**<0.001**
CD19^+^CD27^+^CD24^bright^ regulatory B cells (%); mean (SD)	18.86 (13.25)	35.91 (12.57)	13.38 (7.71)	13.62 (7.87)	**<0.001**
CD19^+^CD27^−^CD24^bright^CD38^bright^ transitional B cells (%); mean (SD)	4.91 (7.70)	2.05 (3.09)	6.34 (8.99)	4.99 (7.53)	**<0.001**
CD19^+^CD20^+^CD138^+^ short-lived plasma cells (%); mean (SD)	0.09 (0.13)	0.08 (0.12)	0.07 (0.11)	0.11 (0.15)	**0.003**
CD19^+^CD20^−^CD138^+^ long-lived plasma cells (%); mean (SD)	0.39 (0.61)	0.65 (1.00)	0.23 (0.29)	0.41 (0.47)	**<0.001**
CD19^+^CD20^+^CD69^+^ activated B cells (%); mean (SD)	0.15 (0.29)	0.19 (0.29)	0.14 (0.31)	0.13 (0.23)	**0.049**
CD19^+^CD38^bright^CD27^bright^ SLE-associated plasma cells (%); mean (SD)	3.37 (4.75)	6.60 (7.03)	1.55 (1.98)	3.56 (4.05)	**<0.001**

Data are presented as numbers (percentage) or means (SD). Statistically significant *P* values are in bold.

Cluster 1 (*n* = 191) was characterized by greater relative proportions cells (mean % ± SD %) of CD19^+^CD27^+^CD24^bright^ regulatory B cells (35.9% ± 12.6%), CD19^+^CD20^+^CD27^+^ bulk memory B cells (32.2% ± 9.9%), CD19^+^CD20^+^CD69^+^ activated B cells (0.2% ± 0.3%), CD19^+^CD20^−^CD138^+^ long-lived plasma cells (0.7% ± 1.0%) and CD19^+^CD38^bright^CD27^bright^ SLE-associated plasma cells (6.6% ± 7.0%) compared with cluster 2 [(13.4% ± 7.7%), (11.4% ± 6.2%), (0.1% ± 0.3%), (0.2% ± 0.3%), (1.6% ± 2.0%), respectively] and cluster 3 [(13.6% ± 7.9%), (11.6% ± 5.9%), (0.1% ± 0.2%), (0.4% ± 0.5%), and (3.6% ± 4.0%), respectively]. In addition, cluster 1 demonstrated a lower proportion of CD19^+^CD20^+^CD27^−^ naïve B cells (56.8% ± 11.5%) and CD19^+^CD27^−^CD24^bright^CD38^bright^ transitional B cells (2.1% ± 3.1%) compared with clusters 2 and 3. Cluster 1 exhibited higher percentages of patients with positive IgM and IgG anticardiolipin antibodies (14.1% and 11.0%, respectively) compared with cluster 2 (4.4% and 2.2%, respectively; *P* < 0.001). Compared with cluster 2, cluster 1 was characterized by overall higher percentages of positive anti-dsDNA (75.9% *vs* 51.9%; *P* < 0.001), anti-Sm (21.2% *vs* 18.0%; *P* < 0.001), low C3 (41.4% *vs* 13.1%; *P* < 0.001) and low C4 (20.1% *vs* 1.6%; *P* < 0.001).

Cluster 2 was the largest cluster, comprising 366 patients. The CD19^+^CD20^+^ B cell pool in this cluster consisted of greater relative proportions of CD19^+^CD27^−^CD24^bright^CD38^bright^ transitional B cells (6.3% ± 9.0%) and CD19^+^CD20^+^CD27^−^ naïve B cells (85.4% ± 7.2%) and lower proportions of CD19^+^CD20^−^CD138^+^ long-lived plasma cells (0.2% ± 0.3%) and CD19^+^CD38^bright^CD27^bright^ SLE-associated plasma cells (1.6% ± 2.0%) compared with cluster 1 [(2.1% ± 3.1%), (56.8% ± 11.5%), (0.7% ± 1.0%), and (6.6% ± 7.0%), respectively] and cluster 3 [(5.0% ± 7.5%), (81.8% ± 9.2%), (0.4% ± 0.5%), and (3.6% ± 4.0%), respectively]. Cluster 2 exhibited overall lower proportions of patients positive for anti-dsDNA (51.9%) and anti-Sm antibodies (18.0%), positive for IgM (4.4%) and IgG anticardiolipin antibodies (2.2%), and with low C3 (13.1%) and low C4 levels (1.6%) compared with cluster 1 (as reported above) and cluster 3 [anti-dsDNA (96.7%) and anti-Sm positivity (55.2%), IgM (17.6%) and IgG (14.2%) anticardiolipin antibody positivity, low C3 (90.4%) and low C4 (67.4%) levels].

Cluster 3 (*n* = 239) was primarily characterized by a higher proportion of relative proportions of CD19^+^CD20^+^CD138^+^ short-lived plasma cells (0.1% ± 0.2%) compared with clusters 1 and 2. Cluster 3 and cluster 2 had comparable relative proportions of B cell subpopulations with regard to CD19^+^CD27^+^CD24^bright^ regulatory B cells, CD19^+^CD20^+^CD27^+^ bulk memory B cells and CD19^+^CD20^+^CD69^+^ activated B cells. Patients in cluster 3 were the most serologically active, with 96.7% of the patients displaying positive anti-dsDNA antibodies, 90.4% and 67.4% low C3 and low C4 levels, respectively, and 55.2% positive anti-Sm antibodies. These frequencies were greater than the respective ones in cluster 1 (75.9%, 41.4%, 20.4%, and 20.9%, respectively; *P* < 0.001 for all) and cluster 2 (51.9%, 13.1%, 1.6%, and 18.0%, respectively; *P* < 0.001 for all). Moreover, the proportions of patients with positive IgG or IgM anticardiolipin antibodies were higher in cluster 3 (14.2% and 17.6%, respectively) than in cluster 2 (2.2% and 4.4%, respectively; *P* < 0.001 for both), but did not differ from those in cluster 1 (11.0%; *P* = 0.502 and 14.1%; *P* = 0.589).

**Figure 2.**
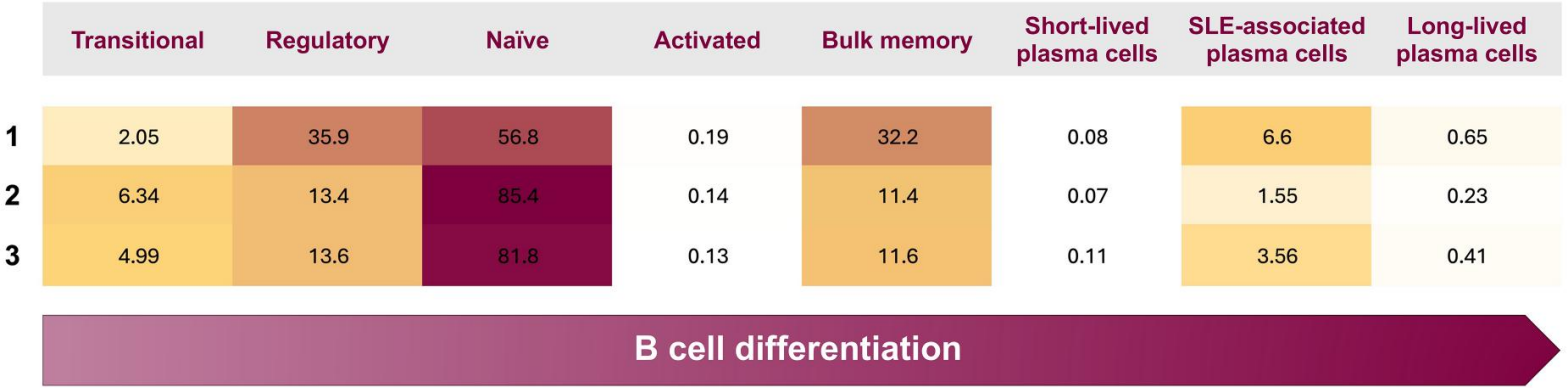
B cell maturation pathway and cluster-specific differences. Schematic representation of the B cell maturation pathway, overlaid with the relative percentages of B cell subsets across the three identified patient clusters. The schematic illustrates key maturation stages of B cells, from transitional and naïve B cells to plasmablasts and long-lived plasma cells, highlighting differences in subset abundance across clusters

We further illustrated the between-cluster differences in B cell subset distribution by mapping them onto a schematic of the B cell maturation pathway, overlaid with the relative abundance of B cell subsets in each cluster (Fig. 2).

### Demographics and treatments

Next, we investigated differences with regard to demographics and treatments across clusters. There was no statistically significant difference across clusters regarding sex distribution or disease duration. Age at baseline differed across the three clusters; patients in cluster 3 were younger (34.3 ± 10.9 years) compared with patients in cluster 1 (38.9 ± 12.5 years; *P* < 0.001) and cluster 2 (41.4 ± 12.4 years; *P* < 0.001). Cluster 3 comprised the lowest proportion of White/Caucasian patients (51.5%) compared with cluster 1 (66.0%; *P* < 0.001) and cluster 2 (62.6%; *P* < 0.001). At baseline, mean SLEDAI-2K scores were higher in cluster 3 (12.2 ± 3.6) than in cluster 2 (9.9 ± 2.7, *P* < 0.001) and cluster 1 (10.4 ± 3.3; *P* < 0.001). The mean prednisone dose at baseline was found to be lower in cluster 2 (9.6 ± 8.6) compared with cluster 1 (12.9 ± 7.8 respectively; *P* < 0.001) and with cluster 3 (11.7 ± 8.1; *P* = 0.006). Use of azathioprine was more common in cluster 1 compared with cluster 2 (33.5% *vs* 13.7%; *P* < 0.001) and with cluster 3 (17.2%; *P* = 0.001). MTX was more commonly used in cluster 2 (13.1%) compared with cluster 1 (6.3%; *P* = 0.044).

The frequency of use of mycophenolic acid was greater among patients in cluster 3 (20.1%) *vs* clusters 1 (5.8%; *P* < 0.001) and *vs* cluster 2 (11.2%; *P* = 0.008). We observed no difference in terms of paired comparison with regard to other background medications.

### Clinical manifestations

**Figure 3.**
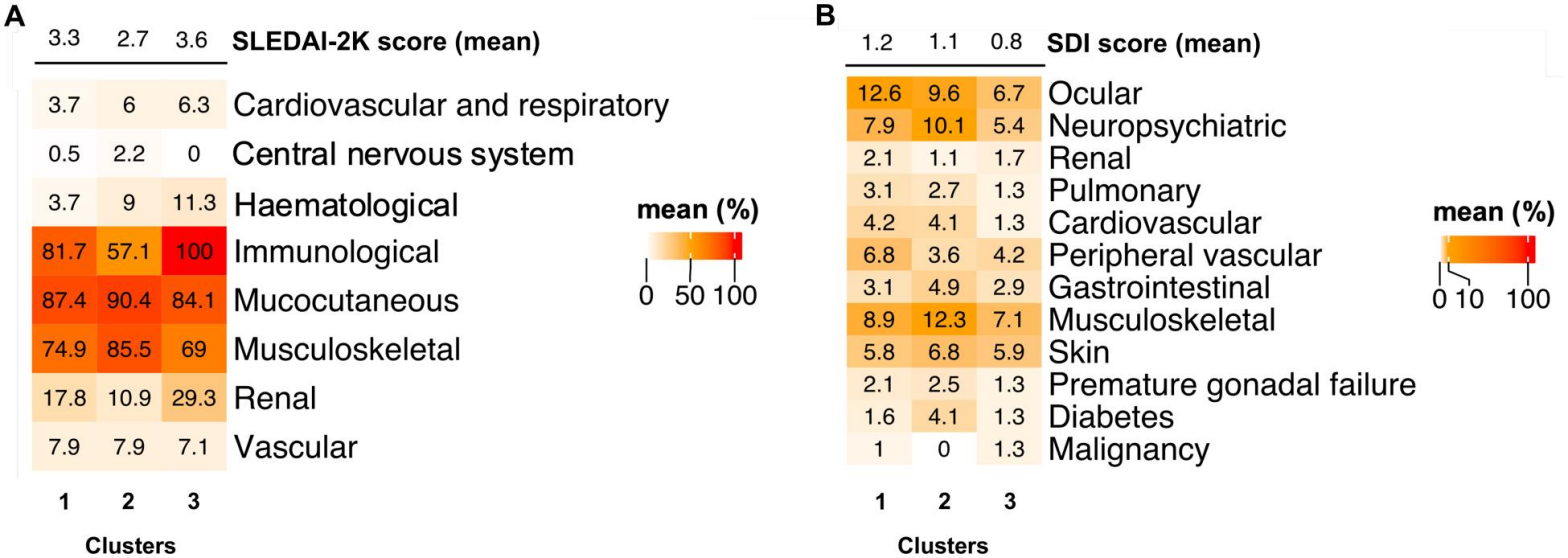
Disease activity and organ damage at baseline by clusters. Distribution of scored (**A**) SLEDAI-2K descriptors and (**B**) SDI items grouped by organ system at baseline, stratified by cluster. SLEDAI-2K: SLEDAI 2000; SDI: SLICC/ACR Damage Index

Fig. 3A details differences across clusters regarding clinical manifestations at baseline. Renal involvement was more common in cluster 3 (29.3%) compared with cluster 2 (10.9%) and cluster 1 (17.8%; *P* < 0.001). The percentage of patients with proteinuria based on SLEDAI-2K differed significantly among the clusters, with the higher percentages of patients with proteinuria observed in cluster 3 compared with clusters 2 and 1 (28.9% *vs* 10.4% *vs* 17.3%, respectively; *P* < 0.001).

We also found a significant difference between cluster 3 and cluster 2 in terms haematuria (5.4% *vs* 1.6%; *P* = 0.035). Additionally, the frequency of leukopenia (10.5%) was higher in cluster 3 when compared with cluster 1 (2.6%; *P* = 0.006). Conversely, we observed that mucocutaneous manifestations were more common in cluster 2 compared with cluster 3 (90.4% *vs* 84.1%; *P* = 0.027). Furthermore, cluster 2 was dominated by musculoskeletal manifestations (85.5%) compared with both cluster 1 (74.9%; *P* = 0.003) and cluster 3 (69.0%; *P* < 0.001). Arthritis was more common in cluster 2 (85.5%) compared with both cluster 1 (74.9%; *P* = 0.003) and cluster 3 (69.0%; *P* < 0.001).

### Organ damage across clusters

Clusters 1 and 2 displayed numerically greater SDI scores compared with cluster 3 at baseline and week 52 (0.67 ± 1.2, 0.69 ± 1.1 and 0.44 ± 0.8 for clusters 1, 2 and 3 at baseline and 0.7 ± 1.3. 0.7 ± 1.1 and 0.48 ± 0.9 at week 52). A higher percentage of cranial and peripheral neuropathy was found in cluster 2 compared with cluster 3 (6.0% *vs* 1.3%; *P* = 0.008) and cluster 1 (1.6%; *P* = 0.029; Fig. 3B).

### Clinical outcomes

**Figure 4.**
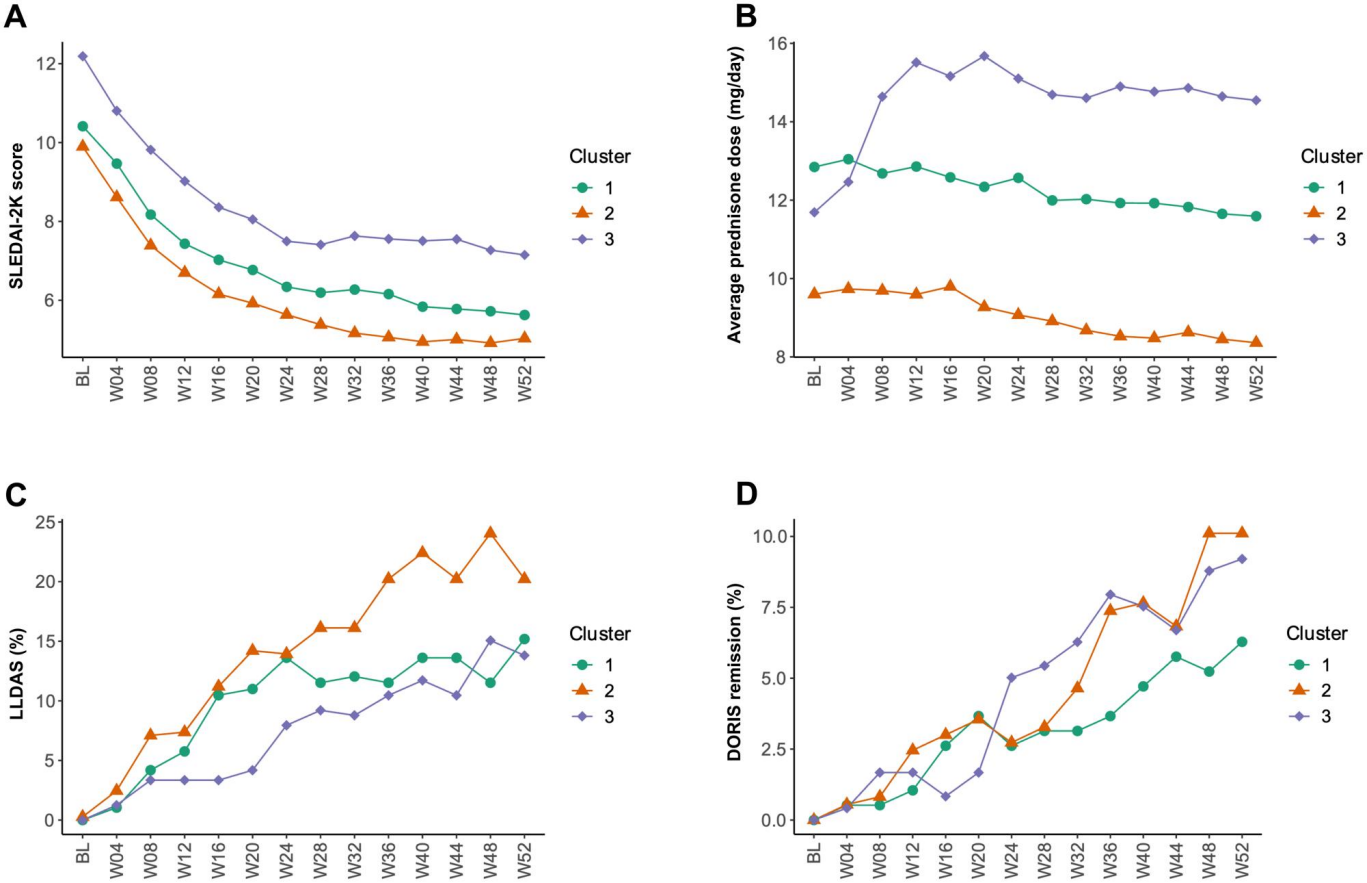
Trajectories of patient clusters over time. Depicted are SLEDAI-2K score trajectories (**A**), mean prednisone equivalent dose (**B**), LLDAS (**C**) and DORIS remission (**D**) from baseline until the end of the follow-up period across clusters. DORIS: definitions of remission in SLE; LLDAS: lupus low disease activity state; SLEDAI-2K: SLEDAI 2000

Next, we examined whether disease activity trajectories from baseline through week 52 differed across the three clusters. Compared with clusters 1 and 3, patients in cluster 2 demonstrated the lowest disease activity assessed using the SLEDAI-2K (Fig. 4A) and were treated with lower doses of glucocorticoids (Fig. 4B). Patients in cluster 2 more frequently were in LLDAS throughout the study period compared with patients in cluster 1 and cluster 3 (Fig. 4C). Cluster 2 also displayed a significantly higher percentage of DORIS remission at weeks 48 and 52 compared with cluster 1 and 3 (Fig. 4D).

### Response to belimumab

**Figure 5.**
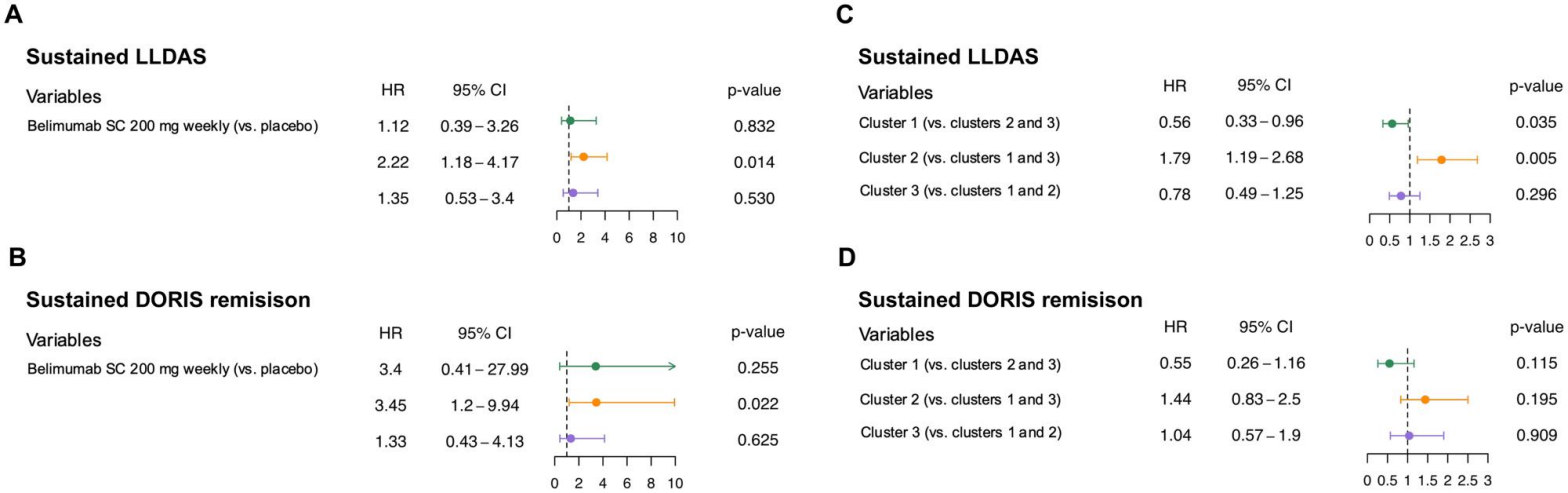
Cox regression applied to the population stratified by clusters. Forest plots illustrating results from Cox regression models investigating clusters in relation to sustained LLDAS (**A**) or sustained DORIS remission (**B**) attainment, adjusting for age, ethnicity, sex and belimumab use, and models investigating belimumab use in relation to sustained LLDAS (**C**) or sustained DORIS remission attainment (**D**) stratified by clusters. DORIS: definitions of remission in SLE; LLDAS: lupus low disease activity state

Cox regression analysis revealed that belimumab was superior to placebo in attaining sustained LLDAS (hazard ratio [HR]: 2.22; 95% CI: 1.18–4.17; *P* = 0.014) and sustained DORIS remission (HR 3.45; 95% CI: 1.2–9.94; *P* = 0.022) in cluster 2. No such association was found in cluster 1 or cluster 3 (Fig. 5A and C).

In multivariate Cox regression analysis, cluster 2 displayed a greater probability of sustained LLDAS compared with cluster 1 and cluster 3 (HR: 1.77; 95% CI: 1.18–2.66; *P* = 0.006). A similar trend was seen for sustained DORIS remission, although not reaching statistical significance (HR: 1.42; 95% CI: 0.82–2.46; *P* = 0.216). Cluster 1 appeared to be negatively associated with attainment of LLDAS compared with cluster 2 and cluster 3 (HR: 0.56; 95% CI: 0.33–0.96; *P* = 0.034; Fig. 5B and D).

## Discussion

Given the central role of B cells and autoantibodies in the pathogenesis of SLE and previous observations that early alterations in specific B cell subsets following initiation of belimumab or placebo are associated with treatment response and flare prevention [12, 14, 15], we hypothesized that B cell immunophenotyping combined with serological profiling could facilitate stratification of patients into distinct clusters. Such stratification might help identify individuals more prone to specific disease manifestations, those requiring closer monitoring or intensified treatment and those more likely to respond favourably to B cell-targeting therapy. Using k-means clustering, we demonstrated that patients segregate into distinct immunological clusters, which differ in their likelihood of achieving LLDAS or DORIS remission, as well as in their response to belimumab treatment.

In this study, we identified three distinct immunological clusters among SLE patients based on B cell and plasma cell subset profiling, each characterized by unique serological and clinical features, disease burden and treatment outcomes. Cluster 2 was defined by a greater proportion of CD19^+^CD27^−^CD24^bright^CD38^bright^ transitional B cells, along with predominant mucocutaneous and musculoskeletal involvement, and more frequent use of MTX. Notably, patients in this cluster showed the greatest benefit from belimumab therapy and were more likely to attain LLDAS and DORIS remission. Cluster 3 exhibited the highest serological activity, including anti-dsDNA, anti-Sm, anticardiolipin antibodies and low complement levels, and was dominated by renal involvement, higher SLEDAI-2K scores and greater use of glucocorticoids and mycophenolic acid. Despite the higher percentage of renal involvement and higher SLEDAI-2K scores in this cluster, prednisone doses were lower than in cluster 2. This likely reflects the eligibility criteria of the BLISS-SC trial, which excluded patients with active severe lupus nephritis, resulting in a cohort where renal involvement was predominantly mild at baseline. Cluster 1 showed higher proportions of CD19^+^CD27^+^CD24^bright^ regulatory B cells and CD19^+^CD20^+^CD27^+^ bulk memory B cells, and fewer CD19^+^CD38^bright^CD27^bright^ SLE-associated plasma cells, representing an intermediate clinical phenotype. Importantly, the associations between cluster assignment and clinical outcomes in our study were significant after adjustment for key covariates, including age, sex, ethnicity and belimumab use, indicating that the observed differences are independent of major confounding factors. This supports the potential utility of B cell immunophenotyping for patient stratification and personalized treatment strategies in SLE.

Unsupervised ML enables the unbiased identification of clusters based on autonomous segregation of variables according to their intrinsic weight [30–32]. Unlike traditional hypothesis-driven statistical approaches, ML uncovers novel associations and patterns within complex datasets [19]. In autoimmune rheumatic diseases, unsupervised clustering has been applied successfully, particularly in rheumatoid arthritis, to identify distinct patient subgroups based on inflammatory pathways despite clinical similarities [33]. Although further validation is required, such strategies offer promising prospects for predicting disease course and treatment response [34]. In SLE, evidence supporting the use of unsupervised clustering is steadily increasing. While most existing studies focus on diagnostic algorithms [19], few have explored its application in predicting treatment response, particularly in lupus nephritis [35, 36]. To our knowledge, this is the first large-scale study to apply unsupervised clustering to immunological profiles of SLE patients undergoing belimumab therapy.

Previous studies have attempted to classify patients with SLE into clusters based primarily on their serological profiles, focusing on association between autoantibodies and specific clinical patterns. For instance, in a cohort study by To *et al.* [37], three autoantibody-based clusters were identified: anti-Sm/anti-RNP antibodies, anti-dsDNA/Ro/La and anti-dsDNA/antiphospholipid antibodies, each linked to distinct clinical phenotypes. The anti-Sm/RNP cluster had a lower prevalence of renal manifestations, while the anti-dsDNA/Ro/La cluster was associated with the highest prevalence of renal SLE. The anti-dsDNA/antiphospholipid cluster was characterized by higher rates of damage, including cerebrovascular events, neuropathy and venous thrombosis. Similarly, a study from South China [38] grouped patients with SLE into three clusters, i.e. anti-Ro/Sm/RNP, anti-Ro alone and absence of anti-ENAs, although no significant differences in renal involvement or anti-dsDNA occurrence were observed across the clusters.

In contrast to these prior studies, our approach focused on baseline B cell profiling rather than solely on serological patterns. B cell alterations likely precede overt changes in autoantibody production and complement consumption, and thus may offer earlier, clinically meaningful stratification of SLE endotypes. Indeed, the clusters identified in our study exhibited distinct clinical trajectories, disease burden, and differential response to belimumab therapy. This provides further support for the notion that variations in underlying immune dysregulation contribute to heterogeneity in disease manifestations and treatment outcomes in SLE [39].

In particular, cluster 2 that was characterized by greater relative proportions of CD19^+^CD27^−^CD24^bright^CD38^bright^ transitional B cells showed a more favourable response to belimumab. This is biologically plausible, given the mechanism of action of belimumab, targeting soluble BAFF and promoting the depletion of immature B cells [40]. Additionally, cluster 2 was enriched for musculoskeletal and cutaneous involvement, disease domains known to be responsive to belimumab [41–44]. However, the proportion of patients with musculoskeletal and skin involvement was not negligible in clusters 2 and 3, suggesting that different molecular profiles lie under similar clinical manifestations, as recently shown on the transcriptional level, likely conferring variable susceptibility to treatment [45].

To aid interpretation, it is important to distinguish between predictors of treatment response and those associated with the attainment of sustained desirable disease states. While patients with high disease activity and serological activity at baseline may show better response according to indices primarily capturing reductions in disease activity, achieving stable low disease activity or remission is often more challenging in patients with more severe disease features, as demonstrated in our study. This likely reflects the underlying disease burden and long-term prognosis. Importantly, the associations between cluster assignment and clinical outcomes were independent of the influence of key covariates, including age, sex and ethnicity. Collectively, our findings suggest that peripheral B cell immunophenotyping may offer a practical approach to stratifying patients with SLE, identifying those who may derive greater benefit from B cell-targeting therapies and potentially guiding personalized treatment strategies.

This study has several limitations. Firstly, the clinical trial setting limits the clinical applicability of the results. To be eligible for participation in BLISS-SC, patients were required to be autoantibody positive (ANA titre ≥1:80 and/or anti-dsDNA ≥30 IU/ml), which may have influenced the results and limit their generalizability. Furthermore, the inclusion criteria did not permit participation of patients with active severe nephritis or CNS disease, so no conclusions can be drawn regarding these patient populations. While further replication and prospective validation are warranted, our results provide a framework for incorporating immunological profiling into clinical decision-making. The identification of distinct immunological endotypes associated with differential treatment response highlights the potential of precision medicine approaches in SLE, where tailoring therapy based on underlying immunopathology may improve outcomes.

In conclusion, k-means clustering identified three immunologically distinct clusters of SLE patients, each associated with differential response to belimumab therapy. These findings suggest that early stratification based on B cell phenotyping and serological profile may help predict disease manifestations and clinical outcomes, enabling clinicians to identify patients with potentially more severe phenotypes and to tailor treatment strategies. Moreover, our results indicate that evaluation of B cell subsets prior to treatment initiation may provide additional guidance in optimizing the use of belimumab. Further studies are warranted to validate these findings and to explore whether the application of ML approaches to complementary data layers, such as the lupus transcriptome, microbiome, metabolome and proteome, may further advance personalized therapeutic strategies in SLE.

## Supplementary Material

keaf215_Supplementary_Data

## Data Availability

The datasets used and analysed during the current study can be made available through the Clinical Study Data Request (CSDR) consortium.
