# Correction to: Unsupervised machine learning identifies distinct SLE patient endotypes with differential response to belimumab

**DOI:** 10.1093/rheumatology/keaf579

**Published:** 2025-11-14

This is a correction to: Roberto Depascale, Raffaele Da Mutten, Julius Lindblom, Nursen Cetrez, Leonardo Palazzo, Luca Iaccarino, Andrea Doria, Dionysis Nikolopoulos, Mariele Gatto, Ioannis Parodis, Unsupervised machine learning identifies distinct SLE patient endotypes with differential response to belimumab, *Rheumatology*, Volume 64, Issue 8, August 2025, Pages 4650–4658, https://doi.org/10.1093/rheumatology/keaf215

In the originally published version of the manuscript, there were errors in Figure 2.

**Figure 2.**
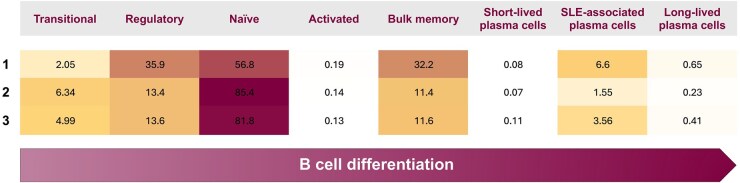
B cell maturation pathway and cluster-specific differences. Schematic representation of the B cell maturation pathway, overlaid with the relative percentages of B cell subsets across the three identified patient clusters. The schematic illustrates key maturation stages of B cells, from transitional and naïve B cells to plasmablasts and long-lived plasma cells, highlighting differences in subset abundance across clusters

The cell types in the headings were not in the correct order. Figure 2 should read:

instead of:

**Figure 2.**
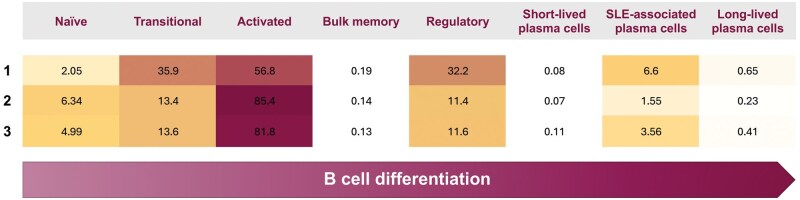
B cell maturation pathway and cluster-specific differences. Schematic representation of the B cell maturation pathway, overlaid with the relative percentages of B cell subsets across the three identified patient clusters. The schematic illustrates key maturation stages of B cells, from transitional and naïve B cells to plasmablasts and long-lived plasma cells, highlighting differences in subset abundance across clusters

The emendation has been made to the article.

