# Supplementary material for: Unsupervised machine learning identifies distinct SLE patient endotypes with differential response to belimumab
Source: Rheumatology (Oxford). 2025 Apr 17;64(8):4650–8. doi: 10.1093/rheumatology/keaf215 (PMC12316369; doi:10.1093/rheumatology/keaf215)
Supplement: keaf215_Supplementary_Data [file keaf215_supplementary_data.zip › rhe-24-2878-File009.pdf]

Unsupervised machine learning identifies distinct systemic lupus erythematosus patient endotypes with differential response to belimumab

Roberto Depascale*, Raffaele Da Mutten*, Julius Lindblom, Nursen Cetrez, Leonardo Palazzo, Luca Iaccarino, Andrea Doria, Dionysis Nikolopoulos, Mariele Gatto**, Ioannis Parodis**

*Contributed equally as first authors

**Contributed equally as senior authors

SUPPLEMENTARY MATERIAL

TABLE OF CONTENTS

Supplementary Table S1. Cluster differences between train and test data. 3

Supplementary Table S2. Clinical and serological data of the study population across clusters. 5

Supplementary Table S3. SLEDAI-2K organ domain involvement at baseline across clusters. 6

Supplementary Table S4. SDI organ domain involvement at baseline across clusters. 7

Supplementary Table S5. SRI-4 response from baseline through week 52 across clusters. 9

Supplementary Table S6. LLDAS attainment from baseline through week 52 across clusters. 10

Supplementary Table S7. DORIS remission attainment from baseline through week 52 across clusters. 11

Supplementary Table S8. cSLEDAI-2K response attainment from baseline through week 52 across clusters. 12

Supplementary Table S9. SLEDAI-2K score from baseline through week 52 across clusters. 13

Supplementary Table S10. Prednisone equivalent dose use from baseline through week 52 across clusters. 14

Supplementary Table S11. Across-cluster and pairwise comparisons regarding demographics and clinical data. 15

Supplementary Table S12. Across-cluster and pairwise comparisons regarding SLEDAI-2K organ domain items. 17

Supplementary Table S13. Across-cluster and pairwise comparisons regarding SDI organ domain items. 18

Supplementary Table S14. Across-cluster and pairwise comparisons regarding SRI-4 response attainment from week 4 through week 52. 20

Supplementary Table S15. Across-cluster and pairwise comparisons regarding LLDAS attainment from baseline through week 52. 21

Supplementary Table S16. Across-cluster and pairwise comparisons regarding DORIS remission attainment from baseline through week 52. 22

Supplementary Table S17. Across-cluster and pairwise comparisons regarding cSLEDAI-2K response attainment from baseline through week 52. 23

Supplementary Table S18. Across-cluster and pairwise comparisons regarding SLEDAI-2K mean score from baseline through week 52. 24

Supplementary Table S19. Across-cluster and pairwise comparisons regarding mean prednisone equivalent dose use from baseline through week 52. 25

Supplementary Table S1. Cluster differences between train and test data.

	All patients	Cluster 1	Cluster 2	Cluster 3	*p* value
Test data	N=239	N=103	N=92	N=44	
Regulatory B cells; mean (SD)	18.39 (13.64)	13.36 (7.39)	13.36 (7.64)	40.70 (12.35)	**<0.001**
Transitional B cells; mean (SD)	4.44 (6.28)	4.69 (5.35)	5.22 (7.49)	2.22 (5.01)	**0.028**
Short-lived plasma cells; mean (SD)	0.08 (0.13)	0.04 (0.08)	0.12 (0.15)	0.08 (0.13)	**<0.001**
Memory B cells; mean (SD)	16.05 (11.38)	12.45 (7.91)	12.73 (7.70)	31.45 (12.07)	**<0.001**
Naïve B cells; mean (SD)	77.97 (15.10)	81.78 (12.81)	81.82 (10.83)	61.00 (16.24)	**<0.001**
Activated B cells; mean (SD)	0.14 (0.21)	0.09 (0.12)	0.19 (0.26)	0.16 (0.24)	**0.003**
Long-lived plasma cells; mean (SD)	0.30 (0.37)	0.26 (0.33)	0.27 (0.28)	0.44 (0.53)	**0.015**
SLE-associated plasma cells; mean (SD)	3.28 (4.51)	2.93 (4.46)	2.91 (3.61)	4.85 (5.88)	**0.037**
Anti-dsDNA (+); n (%)	177 (74.1)	81 (78.6)	66 (71.7)	30 (68.2)	0.337
Anti-cardiolipin IgG (+); n (%)	20 (8.4)	12 (11.7)	4 (4.3)	4 (9.1)	0.181
Anti-cardiolipin IgM (+); n (%)	26 (10.9)	10 (9.7)	7 (7.6)	9 (20.5)	0.070
Anti-Sm (+); n (%)	72 (30.1)	32 (31.1)	33 (35.9)	7 (15.9)	0.058
Low C3; n (%)	110 (46.0)	49 (47.6)	40 (43.5)	21 (47.7)	0.822
Low C4; n (%)	60 (25.1)	28 (27.2)	23 (25.0)	9 (20.5)	0.690
Group; n (%)					**<0.001**
1	103 (43.1)	103 (100.0)	0 (0.0)	0 (0.0)	
2	92 (38.5)	0 (0.0)	92 (100.0)	0 (0.0)	
3	44 (18.4)	0 (0.0)	0 (0.0)	44 (100.0)	
Train data	N=557	N=245	N=144	N=168	
Regulatory B cells; mean (SD)	19.06 (13.09)	13.23 (7.46)	35.48 (11.49)	13.49 (7.90)	**<0.001**
Transitional B cells; mean (SD)	5.11 (8.23)	6.58 (9.63)	2.31 (3.82)	5.37 (8.22)	**<0.001**
Short-lived plasma cells; mean (SD)	0.09 (0.13)	0.08 (0.12)	0.09 (0.12)	0.10 (0.14)	0.315
Memory B cells; mean (SD)	16.61 (11.40)	11.43 (6.35)	31.45 (9.71)	11.46 (6.09)	**<0.001**
Naïve B cells; mean (SD)	77.21 (14.61)	85.47 (7.34)	58.01 (11.03)	81.63 (9.50)	**<0.001**
Activated B cells; mean (SD)	0.15 (0.31)	0.13 (0.32)	0.22 (0.36)	0.13 (0.24)	**0.007**
Long-lived plasma cells; mean (SD)	0.42 (0.68)	0.25 (0.31)	0.70 (1.10)	0.44 (0.52)	**<0.001**
SLE-associated plasma cells; mean (SD)	3.41 (4.86)	1.52 (2.01)	6.20 (6.98)	3.77 (4.34)	**<0.001**
Anti-dsDNA (+); n (%)	389 (69.8)	115 (46.9)	109 (75.7)	165 (98.2)	**<0.001**
Anti-cardiolipin IgG (+); n (%)	43 (7.7)	6 (2.4)	15 (10.4)	22 (13.1)	**<0.001**
Anti-cardiolipin IgM (+); n (%)	59 (10.6)	9 (3.7)	19 (13.2)	31 (18.5)	**<0.001**
Anti-Sm (+); n (%)	166 (29.8)	40 (16.3)	33 (22.9)	93 (55.4)	**<0.001**
Low C3; n (%)	233 (41.8)	27 (11.0)	58 (40.3)	148 (88.1)	**<0.001**
Low C4; n (%)	146 (26.2)	3 (1.2)	33 (22.9)	110 (65.5)	**<0.001**
Group; n (%)					**<0.001**
1	245 (44.0)	245 (100.0)	0 (0.0)	0 (0.0)	
2	144 (25.9)	0 (0.0)	144 (100.0)	0 (0.0)	

3	168 (30.2)	0 (0.0)	0 (0.0)	168 (100.0)

Data are presented as numbers (percentage) or means (standard deviation). Statistically significant *p* values are in bold.

anti-dsDNA: antibodies against double-stranded deoxyribonucleic acid; anti-Sm: anti-Smith antibodies; C3: complement component 3; C4: complement component 4; SD: standard deviation; SLE: systemic lupus erythematosus; (+): positivity.

Supplementary Table S2. Clinical and serological data of the study population across clusters.

	All patients (N=796)	Cluster 1 (N=191)	Cluster 2 (N=366)	Cluster 3 (N=239)	*p* value
Clinical data					
BMI at baseline; mean (SD)	26.06 (6.59)	25.01 (5.47)	27.45 (7.27)	24.78 (5.90)	**<0.001**
SLE disease duration at baseline; median (IQR)	4.40 [1.44–9.07]	4.91 [1.80– 10.78]	3.94 [1.18–8.98]	4.16 [1.62–8.48]	0.243
SLEDAI-2K score at baseline; mean (SD)	10.71 (3.28)	10.41 (3.29)	9.90 (2.72)	12.19 (3.56)	**<0.001**
cSLEDAI-2K score at baseline; mean (SD)	8.34 (3.07)	7.96 (3.09)	8.56 (2.66)	8.31 (3.59)	0.089
PGA score at baseline; mean (SD)	1.57 (0.44)	1.61 (0.41)	1.55 (0.43)	1.59 (0.46)	0.234
Refractoriness to therapy at week 52; n (%)	560 (70.4)	149 (78.0)	226 (61.7)	185 (77.4)	**<0.001**
SDI total score at baseline; mean (SD)	0.61 (1.04)	0.67 (1.23)	0.69 (1.06)	0.44 (0.81)	**0.012**
SDI >1 at baseline; n (%)	110 (13.8)	28 (14.7)	58 (15.8)	24 (10.0)	0.120
SDI total score at week 52; mean (SD)	0.64 (1.08)	0.72 (1.25)	0.71 (1.08)	0.48 (0.91)	**0.020**
Sustained LLDAS; n (%)	101 (12.7)	16 (8.4)	61 (16.7)	24 (10.0)	**0.007**
Sustained DORIS remission; n (%)	53 (6.7)	8 (4.2)	29 (7.9)	16 (6.7)	0.244
Sustained cSLEDAI-2K response; n (%)	212 (26.6)	50 (26.2)	104 (28.4)	58 (24.3)	0.522
Time to sustained LLDAS; mean (SD)	351.31 (45.48)	354.32 (40.96)	346.28 (53.32)	356.62 (33.68)	**0.014**
Time to sustained DORIS remission; mean (SD)	358.18 (31.13)	361.14 (20.87)	357.02 (33.28)	357.58 (34.37)	0.314
Time to cSLEDAI-2K response; mean (SD)	319.73 (90.08)	318.70 (92.49)	317.30 (91.61)	324.28 (85.88)	0.638
Str based on SLEDAI; n (%)	491 (61.7)	108 (56.5)	206 (56.3)	177 (74.1)	**<0.001**
Str based on ethnicity; n (%)	77 (9.7)	12 (6.3)	37 (10.1)	28 (11.7)	0.155
Str based on low complement; n (%)	378 (47.5)	91 (47.6)	54 (14.8)	233 (97.5)	**<0.001**
Serological data					
Anti-dsDNA (+); n (%)	566 (71.1)	145 (75.9)	190 (51.9)	231 (96.7)	**<0.001**
Anti-cardiolipin IgG (+); n (%)	63 (7.9)	21 (11.0)	8 (2.2)	34 (14.2)	**<0.001**
Anti-cardiolipin IgM (+); n (%)	85 (10.7)	27 (14.1)	16 (4.4)	42 (17.6)	**<0.001**
Anti-Sm (+); n (%)	238 (29.9)	40 (20.9)	66 (18.0)	132 (55.2)	**<0.001**
Low C3; n (%)	343 (43.1)	79 (41.4)	48 (13.1)	216 (90.4)	**<0.001**
Low C4; n (%)	206 (25.9)	39 (20.4)	6 (1.6)	161 (67.4)	**<0.001**

Data are presented as numbers (percentage) or means (standard deviation). In case of non-normal distribution, the median (interquartile range) is indicated. Statistically significant *p* values are in bold.

anti-dsDNA: antibodies against double-stranded deoxyribonucleic acid; anti-Sm: anti-Smith antibodies; BMI: body mass index; cSLEDAI-2K: clinical SLEDAI-2K; C3: complement component 3; C4: complement component 4; DORIS: Definitions Of Remission In SLE; PGA: Physician's Global Assessment; IQR: interquartile range; LLDAS: Lupus Low Disease Activity Status; SD: standard deviation; SDI: Systemic Lupus International Collaborating Clinics (SLICC)/American College of Rheumatology (ACR) Damage Index; SLE: systemic lupus erythematosus; SLEDAI-2K: SLE Disease Activity Index 2000; Str: stratification; (+): positivity.

Supplementary Table S3. SLEDAI-2K organ domain involvement at baseline across clusters.

	All patients (N=796)	Cluster 1 (N=191)	Cluster 2 (N=366)	Cluster 3 (N=239)	*p* value
SLEDAI-2K organ domains and items; n (%)					
Neurological domain; n (%)	9 (1.1)	1 (0.5)	8 (2.2)	0 (0.0)	**0.030**
Organic brain syndrome; n (%)	0 (0.0)	0 (0.0)	0 (0.0)	0 (0.0)	NA
Cranial nerve disorders; n (%)	0 (0.0)	0 (0.0)	0 (0.0)	0 (0.0)	NA
Cerebrovascular accident; n (%)	0 (0.0)	0 (0.0)	0 (0.0)	0 (0.0)	NA
Lupus headache; n (%)	6 (0.8)	1 (0.5)	5 (1.4)	0 (0.0)	0.151
Psychosis; n (%)	0 (0.0)	0 (0.0)	0 (0.0)	0 (0.0)	NA
Seizure; n (%)	0 (0.0)	0 (0.0)	0 (0.0)	0 (0.0)	NA
Mucocutaneous domain; n (%)	699 (87.8)	167 (87.4)	331 (90.4)	201 (84.1)	0.065
Alopecia; n (%)	514 (64.6)	121 (63.4)	236 (64.5)	157 (65.7)	0.880
Mucosal ulcers; n (%)	278 (34.9)	60 (31.4)	150 (41.0)	68 (28.5)	**0.003**
Rash; n (%)	561 (70.5)	137 (71.7)	263 (71.9)	161 (67.4)	0.451
Musculoskeletal domain; n (%)	621 (78.0)	143 (74.9)	313 (85.5)	165 (69.0)	**<0.001**
Arthritis; n (%)	621 (78.0)	143 (74.9)	313 (85.5)	165 (69.0)	**<0.001**
Myositis; n (%)	4 (0.5)	1 (0.5)	3 (0.8)	0 (0.0)	0.378
Cardiopulmonary domain; n (%)	44 (5.5)	7 (3.7)	22 (6.0)	15 (6.3)	0.430
Pericarditis; n (%)	9 (1.1)	3 (1.6)	3 (0.8)	3 (1.3)	0.712
Pleurisy; n (%)	37 (4.6)	5 (2.6)	20 (5.5)	12 (5.0)	0.301
Hematological domain; n (%)	67 (8.4)	7 (3.7)	33 (9.0)	27 (11.3)	**0.015**
Leukopenia; n (%)	54 (6.8)	5 (2.6)	24 (6.6)	25 (10.5)	**0.006**
Thrombocytopenia; n (%)	12 (1.5)	4 (2.1)	6 (1.6)	2 (0.8)	0.546
Constitutional domain (fever); n (%)	10 (1.3)	1 (0.5)	5 (1.4)	4 (1.7)	0.549
Renal domain; n (%)	144 (18.1)	34 (17.8)	40 (10.9)	70 (29.3)	**<0.001**
Hematuria; n (%)	23 (2.9)	3 (1.6)	7 (1.9)	13 (5.4)	**0.019**
Pyuria; n (%)	13 (1.6)	2 (1.0)	4 (1.1)	7 (2.9)	0.168
Urinary casts; n (%)	0 (0.0)	0 (0.0)	0 (0.0)	0 (0.0)	NA
Proteinuria; n (%)	140 (17.6)	33 (17.3)	38 (10.4)	69 (28.9)	**<0.001**
Immunological domain; n (%)	604 (75.9)	156 (81.7)	209 (57.1)	239 (100.0)	**<0.001**
Increased DNA binding; n (%)	565 (71.0)	144 (75.4)	190 (51.9)	231 (96.7)	**<0.001**
Low complement; n (%)	377 (47.4)	90 (47.1)	54 (14.8)	233 (97.5)	**<0.001**
Vascular domain; n (%)	61 (7.7)	15 (7.9)	29 (7.9)	17 (7.1)	0.929
Vasculitis; n (%)	61 (7.7)	15 (7.9)	29 (7.9)	17 (7.1)	0.929
Ocular domain (visual disturbance); n (%)	3 (0.4)	0 (0.0)	3 (0.8)	0 (0.0)	0.171

Data are presented as numbers (percentage). Statistically significant *p* values are in bold.

NA: not applicable; SLEDAI-2K: SLE Disease Activity Index 2000.

Supplementary Table S4. SDI organ domain involvement at baseline across clusters.

	All patients (N=796)	Cluster 1 (N=191)	Cluster 2 (N=366)	Cluster 3 (N=239)	*p* value
SDI organ domains and items; n (%)					
Ocular domain; n (%)	75 (9.4)	24 (12.6)	35 (9.6)	16 (6.7)	0.116
Cataract	59 (7.4)	19 (9.9)	30 (8.2)	10 (4.2)	0.057
Retinal change or optic atrophy	20 (2.5)	6 (3.1)	7 (1.9)	7 (2.9)	0.602
Neuropsychiatric domain; (%)	65 (8.2)	15 (7.9)	37 (10.1)	13 (5.4)	0.120
Cognitive impairment	13 (1.6)	1 (0.5)	9 (2.5)	3 (1.3)	0.199
Seizures	7 (0.9)	4 (2.1)	2 (0.5)	1 (0.4)	0.118
Cerebrovascular accident	23 (2.9)	10 (5.2)	7 (1.9)	6 (2.5)	0.078
Cranial or peripheral neuropathy	28 (3.5)	3 (1.6)	22 (6.0)	3 (1.3)	**0.002**
Transverse myelitis	0 (0.0)	0 (0.0)	0 (0.0)	0 (0.0)	NA
Renal domain; n (%)	12 (1.5)	4 (2.1)	4 (1.1)	4 (1.7)	0.634
Estimated or measured GFR <50%	2 (0.3)	2 (1.0)	0 (0.0)	0 (0.0)	**0.042**
Proteinuria (≥3.5g/24h)	9 (1.1)	2 (1.0)	3 (0.8)	4 (1.7)	0.619
ESKD	1 (0.1)	0 (0.0)	1 (0.3)	0 (0.0)	0.555
Pulmonary domain; n (%)	19 (2.4)	6 (3.1)	10 (2.7)	3 (1.3)	0.374
Pulmonary hypertension	7 (0.9)	2 (1.0)	4 (1.1)	1 (0.4)	0.658
Pulmonary fibrosis	10 (1.3)	3 (1.6)	6 (1.6)	1 (0.4)	0.380
Shrinking lung	2 (0.3)	1 (0.5)	0 (0.0)	1 (0.4)	0.416
Pleural fibrosis	1 (0.1)	0 (0.0)	0 (0.0)	1 (0.4)	0.311
Pulmonary infarction	0 (0.0)	0 (0.0)	0 (0.0)	0 (0.0)	NA
Cardiovascular domain; n (%)	26 (3.3)	8 (4.2)	15 (4.1)	3 (1.3)	0.112
Angina or coronary artery bypass	7 (0.9)	2 (1.0)	5 (1.4)	0 (0.0)	0.204
Myocardial infarction	11 (1.4)	4 (2.1)	7 (1.9)	0 (0.0)	0.090
Cardiomyopathy	3 (0.4)	1 (0.5)	2 (0.5)	0 (0.0)	0.524
Valvular disease	5 (0.6)	1 (0.5)	2 (0.5)	2 (0.8)	0.887
Pericarditis or pericardiectomy	6 (0.8)	1 (0.5)	4 (1.1)	1 (0.4)	0.589
Peripheral vascular; n (%)	36 (4.5)	13 (6.8)	13 (3.6)	10 (4.2)	0.205
Claudication	2 (0.3)	1 (0.5)	1 (0.3)	0 (0.0)	0.556
Minor tissue loss	8 (1.0)	2 (1.0)	4 (1.1)	2 (0.8)	0.951
Significant tissue loss	4 (0.5)	1 (0.5)	1 (0.3)	2 (0.8)	0.631
Venous thrombosis	24 (3.0)	10 (5.2)	7 (1.9)	7 (2.9)	0.093
Gastrointestinal domain; n (%)	31 (3.9)	6 (3.1)	18 (4.9)	7 (2.9)	0.385
Infarction or resection of bowel	0 (0.0)	0 (0.0)	0 (0.0)	0 (0.0)	NA
Mesenteric insufficiency	28 (3.5)	6 (3.1)	16 (4.4)	6 (2.5)	0.454
Chronic peritonitis	0 (0.0)	0 (0.0)	0 (0.0)	0 (0.0)	NA
Stricture or upper GI tract surgery	3 (0.4)	0 (0.0)	2 (0.5)	1 (0.4)	0.602
Musculoskeletal domain; n (%)	79 (9.9)	17 (8.9)	45 (12.3)	17 (7.1)	0.098

Muscle atrophy or weakness	17 (2.1)	2 (1.0)	11 (3.0)	4 (1.7)	0.266
Deforming or erosive arthritis	35 (4.4)	9 (4.7)	20 (5.5)	6 (2.5)	0.216
Osteoporosis vertebral collapse	14 (1.8)	5 (2.6)	7 (1.9)	2 (0.8)	0.360
Avascular necrosis	19 (2.4)	3 (1.6)	10 (2.7)	6 (2.5)	0.688
Osteomyelitis	0 (0.0)	0 (0.0)	0 (0.0)	0 (0.0)	NA
Skin domain; n (%)	50 (6.3)	11 (5.8)	25 (6.8)	14 (5.9)	0.840
Scarring chronic alopecia	37 (4.6)	7 (3.7)	21 (5.7)	9 (3.8)	0.403
Extensive scarring of panniculum	12 (1.5)	2 (1.0)	6 (1.6)	4 (1.7)	0.835
Skin ulceration	7 (0.9)	3 (1.6)	2 (0.5)	2 (0.8)	0.468
Diabetes regardless of therapy; n (%)	16 (2.0)	4 (2.1)	9 (2.5)	3 (1.3)	0.585
Premature gonadal failure; n (%)	21 (2.6)	3 (1.6)	15 (4.1)	3 (1.3)	0.059
Malignancy (excluding dysplasia); n (%)	5 (0.6)	2 (1.0)	0 (0.0)	3 (1.3)	0.113

Data are presented as numbers (percentage). Statistically significant *p* values are in bold.

NA: not applicable; SDI: Systemic Lupus International Collaborating Clinics (SLICC)/American College of Rheumatology (ACR) Damage Index.

Supplementary Table S5. SRI-4 response from baseline through week 52 across clusters.

	All patients (N=796)	Cluster 1 (N=191)	Cluster 2 (N=366)	Cluster 3 (N=239)	*p* value
SRI-4 response; n (%)					
SRI-4 at baseline	0 (0.0)	0 (0.0)	0 (0.0)	0 (0.0)	NA
SRI-4 at week 4	169 (21.3)	33 (17.3)	77 (21.1)	59 (24.9)	0.159
SRI-4 at week 8	292 (36.8)	67 (35.1)	136 (37.3)	89 (37.6)	0.846
SRI-4 at week 12	359 (45.3)	89 (46.6)	167 (45.8)	103 (43.5)	0.785
SRI-4 at week 16	423 (53.3)	100 (52.4)	200 (54.8)	123 (51.9)	0.747
SRI-4 at week 20	438 (55.2)	105 (55.0)	203 (55.6)	130 (54.9)	0.980
SRI-4 at week 24	449 (56.6)	110 (57.6)	202 (55.3)	137 (57.8)	0.798
SRI-4 at week 28	468 (59.0)	107 (56.0)	218 (59.7)	143 (60.3)	0.620
SRI-4 at week 32	454 (57.3)	104 (54.5)	216 (59.2)	134 (56.5)	0.545
SRI-4 at week 36	457 (57.6)	107 (56.0)	217 (59.5)	133 (56.1)	0.631
SRI-4 at week 40	462 (58.3)	118 (61.8)	213 (58.4)	131 (55.3)	0.398
SRI-4 at week 44	452 (57.0)	114 (59.7)	212 (58.1)	126 (53.2)	0.340
SRI-4 at week 48	461 (58.1)	115 (60.2)	211 (57.8)	135 (57.0)	0.784
SRI-4 at week 52	455 (57.4)	118 (61.8)	201 (55.1)	136 (57.4)	0.315

Data are presented as numbers (percentage). Statistically significant *p* values are in bold.

NA: not applicable; SRI-4: SLE responder index 4.

Supplementary Table S6. LLDAS attainment from baseline through week 52 across clusters.

	All patients (N=796)	Cluster 1 (N=191)	Cluster 2 (N=366)	Cluster 3 (N=239)	*p* value
LLDAS attainment; n (%)					
LLDAS at baseline	1 (0.1)	0 (0.0)	1 (0.3)	0 (0.0)	0.555
LLDAS at week 4	14 (1.8)	2 (1.0)	9 (2.5)	3 (1.3)	0.377
LLDAS at week 8	42 (5.3)	8 (4.2)	26 (7.1)	8 (3.3)	0.096
LLDAS at week 12	46 (5.8)	11 (5.8)	27 (7.4)	8 (3.3)	0.116
LLDAS at week 16	69 (8.7)	20 (10.5)	41 (11.2)	8 (3.3)	**0.002**
LLDAS at week 20	83 (10.4)	21 (11.0)	52 (14.2)	10 (4.2)	**<0.001**
LLDAS at week 24	96 (12.1)	26 (13.6)	51 (13.9)	19 (7.9)	0.065
LLDAS at week 28	103 (12.9)	22 (11.5)	59 (16.1)	22 (9.2)	**0.037**
LLDAS at week 32	103 (12.9)	23 (12.0)	59 (16.1)	21 (8.8)	**0.029**
LLDAS at week 36	121 (15.2)	22 (11.5)	74 (20.2)	25 (10.5)	**0.001**
LLDAS at week 40	136 (17.1)	26 (13.6)	82 (22.4)	28 (11.7)	**0.001**
LLDAS at week 44	125 (15.7)	26 (13.6)	74 (20.2)	25 (10.5)	**0.004**
LLDAS at week 48	146 (18.3)	22 (11.5)	88 (24.0)	36 (15.1)	**<0.001**
LLDAS at week 52	136 (17.1)	29 (15.2)	74 (20.2)	33 (13.8)	0.089

Data are presented as numbers (percentage). Statistically significant *p* values are in bold.

LLDAS: Lupus Low Disease Activity Status.

Supplementary Table S7. DORIS remission attainment from baseline through week 52 across clusters.

	All patients (N=796)	Cluster 1 (N=191)	Cluster 2 (N=366)	Cluster 3 (N=239)	*p* value
DORIS remission; n (%)					
Remission at baseline	0 (0.0)	0 (0.0)	0 (0.0)	0 (0.0)	NA
Remission at week 4	4 (0.5)	1 (0.5)	2 (0.5)	1 (0.4)	0.975
Remission at week 8	8 (1.0)	1 (0.5)	3 (0.8)	4 (1.7)	0.439
Remission at week 12	15 (1.9)	2 (1.0)	9 (2.5)	4 (1.7)	0.488
Remission at week 16	18 (2.3)	5 (2.6)	11 (3.0)	2 (0.8)	0.200
Remission at week 20	24 (3.0)	7 (3.7)	13 (3.6)	4 (1.7)	0.349
Remission at week 24	27 (3.4)	5 (2.6)	10 (2.7)	12 (5.0)	0.250
Remission at week 28	31 (3.9)	6 (3.1)	12 (3.3)	13 (5.4)	0.336
Remission at week 32	38 (4.8)	6 (3.1)	17 (4.6)	15 (6.3)	0.314
Remission at week 36	53 (6.7)	7 (3.7)	27 (7.4)	19 (7.9)	0.157
Remission at week 40	55 (6.9)	9 (4.7)	28 (7.7)	18 (7.5)	0.389
Remission at week 44	52 (6.5)	11 (5.8)	25 (6.8)	16 (6.7)	0.882
Remission at week 48	68 (8.5)	10 (5.2)	37 (10.1)	21 (8.8)	0.146
Remission at week 52	71 (8.9)	12 (6.3)	37 (10.1)	22 (9.2)	0.317

Data are presented as numbers (percentage). Statistically significant *p* values are in bold.

DORIS: Definitions Of Remission In SLE; NA: not applicable.

Supplementary Table S8. cSLEDAI-2K response attainment from baseline through week 52 across clusters.

	All patients (N=796)	Cluster 1 (N=191)	Cluster 2 (N=366)	Cluster 3 (N=239)	*p* value
cSLEDAI-2K response; n (%)					
cSLEDAI-2K at baseline	3 (0.4)	1 (0.5)	1 (0.3)	1 (0.4)	0.894
cSLEDAI-2K at week 4	28 (3.5)	4 (2.1)	15 (4.1)	9 (3.8)	0.461
cSLEDAI-2K at week 8	67 (8.4)	19 (9.9)	30 (8.2)	18 (7.5)	0.655
cSLEDAI-2K at week 12	101 (12.7)	26 (13.6)	48 (13.1)	27 (11.3)	0.732
cSLEDAI-2K at week 16	137 (17.2)	36 (18.8)	66 (18.0)	35 (14.6)	0.441
cSLEDAI-2K at week 20	158 (19.8)	40 (20.9)	71 (19.4)	47 (19.7)	0.907
cSLEDAI-2K at week 24	178 (22.4)	44 (23.0)	77 (21.0)	57 (23.8)	0.696
cSLEDAI-2K at week 28	210 (26.4)	45 (23.6)	96 (26.2)	69 (28.9)	0.461
cSLEDAI-2K at week 32	212 (26.6)	47 (24.6)	102 (27.9)	63 (26.4)	0.706
cSLEDAI-2K at week 36	223 (28.0)	52 (27.2)	104 (28.4)	67 (28.0)	0.957
cSLEDAI-2K at week 40	227 (28.5)	56 (29.3)	107 (29.2)	64 (26.8)	0.776
cSLEDAI-2K at week 44	229 (28.8)	58 (30.4)	104 (28.4)	67 (28.0)	0.851
cSLEDAI-2K at week 48	245 (30.8)	57 (29.8)	116 (31.7)	72 (30.1)	0.874
cSLEDAI-2K at week 52	244 (30.7)	60 (31.4)	114 (31.1)	70 (29.3)	0.859

Data are presented as numbers (percentage). Statistically significant *p* values are in bold.

cSLEDAI: clinical SLE disease activity index 2000.

Supplementary Table S9. SLEDAI-2K score from baseline through week 52 across clusters.

	All patients (N=796)	Cluster 1 (N=191)	Cluster 2 (N=366)	Cluster 3 (N=239)	*p* value
SLEDAI-2K score; mean (SD)					
SLEDAI-2K at baseline	10.71 (3.28)	10.41 (3.29)	9.90 (2.72)	12.19 (3.56)	**<0.001**
SLEDAI-2K at week 4	9.48 (3.77)	9.47 (3.61)	8.61 (3.40)	10.80 (4.05)	**<0.001**
SLEDAI-2K at week 8	8.31 (3.98)	8.17 (3.99)	7.39 (3.58)	9.82 (4.13)	**<0.001**
SLEDAI-2K at week 12	7.57 (4.06)	7.43 (3.93)	6.70 (3.77)	9.02 (4.21)	**<0.001**
SLEDAI-2K at week 16	7.03 (4.07)	7.03 (4.40)	6.16 (3.72)	8.36 (3.95)	**<0.001**
SLEDAI-2K at week 20	6.77 (4.06)	6.77 (4.10)	5.93 (3.66)	8.05 (4.27)	**<0.001**
SLEDAI-2K at week 24	6.36 (3.86)	6.34 (3.91)	5.64 (3.53)	7.50 (4.06)	**<0.001**
SLEDAI-2K at week 28	6.19 (4.02)	6.19 (3.91)	5.38 (3.66)	7.41 (4.32)	**<0.001**
SLEDAI-2K at week 32	6.17 (4.26)	6.27 (4.29)	5.17 (3.80)	7.63 (4.48)	**<0.001**
SLEDAI-2K at week 36	6.07 (4.22)	6.16 (4.06)	5.06 (3.63)	7.56 (4.73)	**<0.001**
SLEDAI-2K at week 40	5.93 (4.26)	5.84 (4.12)	4.95 (3.61)	7.51 (4.79)	**<0.001**
SLEDAI-2K at week 44	5.96 (4.31)	5.78 (4.25)	5.01 (3.61)	7.55 (4.87)	**<0.001**
SLEDAI-2K at week 48	5.82 (4.22)	5.72 (4.01)	4.92 (3.68)	7.27 (4.73)	**<0.001**
SLEDAI-2K at week 52	5.81 (4.30)	5.63 (4.19)	5.04 (3.85)	7.15 (4.72)	**<0.001**

Data are presented as mean (standard deviation). Statistically significant *p* values are in bold.

SD: standard deviation. SLEDAI-2K: SLE disease activity index 2000.

Supplementary Table S10. Prednisone equivalent dose use from baseline through week 52 across clusters.

	All patients (N=796)	Cluster 1 (N=191)	Cluster 2 (N=366)	Cluster 3 (N=239)	*p* value
Prednisone equivalent dose; mean (SD)					
Prednisone equivalent dose at baseline	11.01 (8.49)	12.85 (7.78)	9.60 (8.85)	11.69 (8.14)	<0.001
Prednisone equivalent dose at week 4	11.35 (9.07)	13.04 (7.75)	9.73 (9.16)	12.46 (9.53)	<0.001
Prednisone equivalent dose at week 8	11.89 (16.25)	12.68 (7.80)	9.69 (9.16)	14.64 (26.25)	0.001
Prednisone equivalent dose at week 12	12.15 (17.43)	12.86 (8.97)	9.59 (8.81)	15.51 (28.46)	<0.001
Prednisone equivalent dose at week 16	12.07 (17.59)	12.58 (8.56)	9.79 (9.88)	15.16 (28.43)	0.001
Prednisone equivalent dose at week 20	11.93 (17.65)	12.34 (8.34)	9.27 (8.72)	15.68 (29.03)	<0.001
Prednisone equivalent dose at week 24	11.72 (18.32)	12.57 (9.27)	9.07 (8.56)	15.10 (30.28)	<0.001
Prednisone equivalent dose at week 28	11.39 (18.17)	11.99 (8.51)	8.91 (8.38)	14.69 (30.28)	0.001
Prednisone equivalent dose at week 32	11.26 (18.17)	12.02 (8.47)	8.68 (8.29)	14.61 (30.30)	<0.001
Prednisone equivalent dose at week 36	11.26 (18.67)	11.93 (8.36)	8.53 (8.25)	14.90 (31.29)	<0.001
Prednisone equivalent dose at week 40	11.19 (18.69)	11.92 (8.38)	8.48 (8.30)	14.77 (31.32)	<0.001
Prednisone equivalent dose at week 44	11.27 (18.94)	11.82 (8.26)	8.63 (9.50)	14.86 (31.33)	<0.001
Prednisone equivalent dose at week 48	11.08 (18.94)	11.65 (8.28)	8.45 (9.46)	14.64 (31.35)	<0.001
Prednisone equivalent dose at week 52	10.99 (18.95)	11.59 (8.29)	8.36 (9.45)	14.55 (31.37)	<0.001

Data are presented as mean (standard deviation). Statistically significant *p* values are in bold.

SD: standard deviation.

Supplementary Table S11. Across-cluster and pairwise comparisons regarding demographics and clinical data.

	Overall comparison	Pairwise cluster 1 versus cluster 2	Pairwise cluster 1 versus cluster 3	Pairwise cluster 2 versus cluster 3
Demographics				
Age	**<0.001**	0.075	**<0.001**	**<0.001**
Female sex	0.532	0.758	0.776	0.353
Ethnicity				
Asian	**0.036**	0.983	0.072	**0.020**
Black/African American	0.155	0.175	0.078	0.625
Indigenous American	0.887	1.000	0.828	0.772
White/Caucasian	**0.004**	0.484	**0.003**	**0.009**
Clinical data				
BMI at baseline	**<0.001**	**<0.001**	0.926	**<0.001**
SLE disease duration at baseline	0.175	0.361	0.157	0.771
SLEDAI-2K score at baseline	**<0.001**	0.154	**<0.001**	**<0.001**
cSLEDAI-2K score at baseline	0.089	0.073	0.484	0.571
PGA score at baseline	0.234	0.254	0.898	0.466
Refractoriness to therapy at week 52	**<0.001**	**<0.001**	0.974	**<0.001**
SDI total score at baseline	**0.012**	0.979	0.064	**0.013**
SDI >1 at baseline	0.120	0.807	0.190	0.055
SDI total score at week 52	**0.020**	0.991	0.055	**0.029**
Sustained LLDAS	**0.007**	**0.010**	0.672	**0.030**
Sustained DORIS remission	0.244	0.133	0.361	0.686
Sustained cSLEDAI-2K response	0.522	0.645	0.732	0.302
Time to sustained LLDAS	**0.014**	0.115	0.861	**0.017**
Time to sustained DORIS remission	0.314	0.301	0.467	0.975
Time to cSLEDAI-2K response	0.638	0.983	0.799	0.621
Str based on SLEDAI	**<0.001**	1.000	**<0.001**	**<0.001**
Str based on ethnicity	0.155	0.175	0.078	0.625
Str based on low complement	**<0.001**	**<0.001**	**<0.001**	**<0.001**
Medications				
Antimalarial agents	0.162	0.074	0.467	0.344
Azathioprine	**<0.001**	**<0.001**	**<0.001**	0.290
Methotrexate	**0.046**	**0.020**	0.173	0.394
Mofetil mycophenolate	**<0.001**	0.052	**<0.001**	0.004
Cyclophosphamide	0.460	0.565	0.845	1.000
Tacrolimus	0.163	0.110	0.142	1.000
Cyclosporine	0.678	0.861	1.000	0.599
Leflunomide	0.378	0.855	0.845	0.325

Belimumab 200 mg subcutaneous	0.704	0.755	0.467	0.657

Comparisons were performed using the one-way analysis of variance (ANOVA) for continuous variables and Pearson's chi-squared test (χ^2) for categorical variables. Statistically significant p values are in bold.

BMI: body mass index; cSLEDAI-2K: clinical SLEDAI-2K; DORIS: Definitions Of Remission In SLE; PGA: Physician's Global Assessment; LLDAS: Lupus Low Disease Activity Status; SDI: Systemic Lupus International Collaborating Clinics (SLICC)/American College of Rheumatology (ACR) Damage Index; SLE: systemic lupus erythematosus; SLEDAI-2K: SLE Disease Activity Index 2000; (+): positivity.

Supplementary Table S12. Across-cluster and pairwise comparisons regarding SLEDAI-2K organ domain items.

	Overall comparison	Pairwise cluster 1 versus cluster 2	Pairwise cluster 1 versus cluster 3	Pairwise cluster 2 versus cluster 3
SLEDAI-2K organ domains and items				
Neurological domain	**0.030**	0.261	0.910	0.053
Organic brain syndrome	**<0.001**	**<0.001**	**0.021**	**<0.001**
Cranial nerve disorders	**<0.001**	**<0.001**	**0.021**	**<0.001**
Cerebrovascular accident	**<0.001**	**<0.001**	**0.021**	**<0.001**
Lupus headache	0.151	0.630	0.910	0.175
Psychosis	**<0.001**	**<0.001**	**0.021**	**<0.001**
Seizure	**<0.001**	**<0.001**	**0.021**	**<0.001**
Mucocutaneous domain	0.065	0.343	0.401	**0.027**
Alopecia	0.880	0.864	0.687	0.828
Mucosal ulcers	**0.003**	**0.034**	0.575	**0.002**
Rash	0.451	1.000	0.385	0.276
Musculoskeletal domain	**<0.001**	**0.003**	0.221	**<0.001**
Arthritis	**<0.001**	**0.003**	0.221	**<0.001**
Myositis	0.378	1.000	0.910	0.417
Cardiopulmonary domain	0.430	0.326	0.317	1.000
Pericarditis	0.712	0.702	1.000	0.913
Pleurisy	0.301	0.185	0.307	0.958
Hematological domain	**0.015**	**0.032**	**0.006**	0.436
Leukopenia	**0.006**	0.074	**0.003**	0.117
Thrombocytopenia	0.546	0.962	0.490	0.631
Constitutional domain (fever)	0.549	0.630	0.514	1.000
Renal domain	**<0.001**	**0.033**	**0.008**	**<0.001**
Hematuria	**0.019**	1.000	0.064	**0.032**
Pyuria	0.168	1.000	0.310	0.180
Urinary casts	**<0.001**	**<0.001**	**0.021**	**<0.001**
Proteinuria	**<0.001**	**0.029**	**0.007**	**<0.001**
Immunological domain	**<0.001**	**<0.001**	**<0.001**	**<0.001**
Increased DNA binding	**<0.001**	**<0.001**	**<0.001**	**<0.001**
Low complement	**<0.001**	**<0.001**	**<0.001**	**<0.001**
Vascular domain	0.929	1.000	0.916	0.833
Vasculitis	0.929	1.000	0.916	0.833
Ocular domain (visual disturbance)	0.171	0.519	0.021	0.417

Comparisons are performed using the Chi squared test. Statistically significant *p* values are in bold.
SLEDAI-2K: SLE disease activity index 2000.

Supplementary Table S13. Across-cluster and pairwise comparisons regarding SDI organ domain items.

	Overall comparison	Pairwise cluster 1 versus cluster 2	Pairwise cluster 1 versus cluster 3	Pairwise cluster 2 versus cluster 3
SDI organ domains and items				
Ocular domain	0.116	0.343	0.055	0.275
Cataract	0.057	0.593	**0.030**	0.076
Retinal change or optic atrophy	0.602	0.538	1.000	0.592
Neuropsychiatric domain	0.120	0.474	0.417	0.059
Cognitive impairment	0.199	0.195	0.780	0.459
Seizures	0.118	0.212	0.247	1.000
Cerebrovascular accident	0.078	0.057	0.220	0.834
Cranial or peripheral neuropathy	**0.002**	**0.029**	1.000	**0.008**
Transverse myelitis	**<0.001**	**<0.001**	**0.021**	**<0.001**
Renal domain	0.634	0.570	1.000	0.805
Estimated or measured GFR <50%	**0.042**	0.224	0.383	**<0.001**
Proteinuria (≥3.5g/24h)	0.619	1.000	0.891	0.568
ESKD	0.555	1.000	**0.021**	1.000
Pulmonary domain	0.374	0.994	0.308	0.348
Pulmonary hypertension	0.658	1.000	0.845	0.662
Pulmonary fibrosis	0.380	1.000	0.465	0.325
Shrinking lung	0.416	0.740	1.000	0.830
Pleural fibrosis	0.311	**<0.001**	1.000	0.830
Pulmonary infarction	**<0.001**	**<0.001**	**0.021**	**<0.001**
Cardiovascular domain	0.112	1.000	0.108	0.077
Angina or coronary artery bypass	0.204	1.000	0.383	0.175
Myocardial infarction	0.090	1.000	0.081	0.078
Cardiomyopathy	0.524	1.000	0.910	0.674
Valvular disease	0.887	1.000	1.000	1.000
Pericarditis or pericardiectomy	0.589	0.839	1.000	0.662
Peripheral vascular	0.205	0.129	0.325	0.857
Claudication	0.556	1.000	0.910	1.000
Minor tissue loss	0.951	1.000	1.000	1.000
Significant tissue loss	0.631	1.000	1.000	0.709
Venous thrombosis	0.093	0.057	0.332	0.592
Gastrointestinal domain	0.385	0.447	1.000	0.321
Infarction or resection of bowel	**<0.001**	**<0.001**	**0.021**	**<0.001**
Mesenteric insufficiency	0.454	0.632	0.920	0.330
Chronic peritonitis	**<0.001**	**<0.001**	**0.021**	**<0.001**
Stricture or upper GI tract surgery	0.602	0.782	1.000	1.000

Musculoskeletal domain	0.098	0.286	0.615	0.055
Muscle atrophy or weakness	0.266	0.247	0.891	0.446
Deforming or erosive arthritis	0.216	0.858	0.331	0.122
Osteoporosis vertebral collapse	0.360	0.813	0.286	0.468
Avascular necrosis	0.688	0.571	0.736	1.000
Osteomyelitis	**<0.001**	**<0.001**	**0.021**	**<0.001**
Skin domain	0.840	0.759	1.000	0.759
Scarring chronic alopecia	0.403	0.391	1.000	0.368
Extensive scarring of panniculum	0.835	0.855	0.891	1.000
Skin ulceration	0.468	0.457	0.801	1.000
Diabetes regardless of therapy	0.585	1.000	0.764	0.459
Premature gonadal failure	0.059	0.177	1.000	0.077
Malignancy (excluding dysplasia)	0.113	0.224	1.000	0.120

Comparisons are performed using the Pearson's chi-squared test (χ^2). Statistically significant p values are in bold. SDI: Systemic Lupus International Collaborating Clinics (SLICC)/American College of Rheumatology (ACR) Damage Index.

Supplementary Table S14. Across-cluster and pairwise comparisons regarding SRI-4 response attainment from week 4 through week 52.

	Overall comparison	Pairwise cluster 1 versus cluster 2	Pairwise cluster 1 versus cluster 3	Pairwise cluster 2 versus cluster 3
SRI-4 response				
SRI-4 at week 4	0.159	0.336	0.074	0.323
SRI-4 at week 8	0.846	0.678	0.669	1.000
SRI-4 at week 12	0.785	0.920	0.582	0.639
SRI-4 at week 16	0.747	0.647	1.000	0.540
SRI-4 at week 20	0.980	0.956	1.000	0.920
SRI-4 at week 24	0.798	0.676	1.000	0.609
SRI-4 at week 28	0.620	0.452	0.423	0.949
SRI-4 at week 32	0.545	0.327	0.738	0.578
SRI-4 at week 36	0.631	0.491	1.000	0.468
SRI-4 at week 40	0.398	0.490	0.208	0.508
SRI-4 at week 44	0.340	0.784	0.210	0.270
SRI-4 at week 48	0.784	0.649	0.563	0.904
SRI-4 at week 52	0.315	0.153	0.411	0.635

Comparisons are performed using the Pearson's chi-squared test (χ^2). Statistically significant p values are in bold. SRI-4: SLE Responder Index 4.

Supplementary Table S15. Across-cluster and pairwise comparisons regarding LLDAS attainment from baseline through week 52.

	Overall comparison	Pairwise cluster 1 versus cluster 2	Pairwise cluster 1 versus cluster 3	Pairwise cluster 2 versus cluster 3
LLDAS attainment				
LLDAS at baseline	0.555	1.000	0.021	1.000
LLDAS at week 4	0.377	0.414	1.000	0.459
LLDAS at week 8	0.096	0.239	0.840	0.075
LLDAS at week 12	0.116	0.588	0.331	0.058
LLDAS at week 16	**0.002**	0.905	**0.005**	**<0.001**
LLDAS at week 20	**<0.001**	0.350	**0.012**	**<0.001**
LLDAS at week 24	0.065	1.000	0.081	**0.034**
LLDAS at week 28	**0.037**	0.182	0.531	**0.020**
LLDAS at week 32	**0.029**	0.245	0.344	**0.013**
LLDAS at week 36	**0.001**	**0.014**	0.846	**0.002**
LLDAS at week 40	**0.001**	**0.017**	0.657	**0.001**
LLDAS at week 44	**0.004**	0.070	0.393	**0.002**
LLDAS at week 48	**<0.001**	**<0.001**	0.354	**0.010**
LLDAS at week 52	0.089	0.181	0.791	0.056

Comparisons are performed using the Pearson's chi-squared test (χ^2). Statistically significant p values are in bold.
LLDAS: Lupus Low Disease Activity Status.

Supplementary Table S16. Across-cluster and pairwise comparisons regarding DORIS remission attainment from baseline through week 52.

	Overall comparison	Pairwise cluster 1 versus cluster 2	Pairwise cluster 1 versus cluster 3	Pairwise cluster 2 versus cluster 3
DORIS remission				
Remission at baseline	**<0.001**	**<0.001**	**0.021**	**<0.001**
Remission at week 4	0.975	1.000	1.000	1.000
Remission at week 8	0.439	1.000	0.514	0.568
Remission at week 12	0.488	0.414	0.891	0.715
Remission at week 16	0.200	1.000	0.286	0.131
Remission at week 20	0.349	1.000	0.321	0.265
Remission at week 24	0.250	1.000	0.307	0.212
Remission at week 28	0.336	1.000	0.360	0.273
Remission at week 32	0.314	0.534	0.203	0.490
Remission at week 36	0.157	0.121	0.099	0.918
Remission at week 40	0.389	0.253	0.319	1.000
Remission at week 44	0.882	0.759	0.844	1.000
Remission at week 48	0.146	0.071	0.220	0.690
Remission at week 52	0.317	0.175	0.349	0.821

Comparisons are performed using the Pearson's chi-squared test (χ^2). Statistically significant p values are in bold. DORIS: Definitions Of Remission In SLE.

Supplementary Table S17. Across-cluster and pairwise comparisons regarding cSLEDAI-2K response attainment from baseline through week 52.

	Overall comparison	Pairwise cluster 1 versus cluster 2	Pairwise cluster 1 versus cluster 3	Pairwise cluster 2 versus cluster 3
cSLEDAI-2K response				
cSLEDAI-2K response at baseline	0.894	1.000	1.000	1.000
cSLEDAI-2K response at week 4	0.461	0.322	0.470	1.000
cSLEDAI-2K response at week 8	0.655	0.593	0.475	0.887
cSLEDAI-2K response at week 12	0.732	0.974	0.563	0.591
cSLEDAI-2K response at week 16	0.441	0.904	0.300	0.327
cSLEDAI-2K response at week 20	0.907	0.748	0.836	1.000
cSLEDAI-2K response at week 24	0.696	0.664	0.934	0.475
cSLEDAI-2K response at week 28	0.461	0.558	0.259	0.536
cSLEDAI-2K response at week 32	0.706	0.469	0.762	0.753
cSLEDAI-2K response at week 36	0.957	0.843	0.938	0.992
cSLEDAI-2K response at week 40	0.776	1.000	0.634	0.573
cSLEDAI-2K response at week 44	0.851	0.702	0.673	0.992
cSLEDAI-2K response at week 48	0.874	0.725	1.000	0.751
cSLEDAI-2K response at week 52	0.859	1.000	0.711	0.692

Comparisons are performed using the Pearson's chi-squared test (χ^2). Statistically significant *p* values are in bold. cSLEDAI-2K: clinical SLEDAI 2000.

Supplementary Table S18. Across-cluster and pairwise comparisons regarding SLEDAI-2K mean score from baseline through week 52.

	Overall comparison	Pairwise cluster 1 versus cluster 2	Pairwise cluster 1 versus cluster 3	Pairwise cluster 2 versus cluster 3
SLEDAI-2K score				
SLEDAI-2K at baseline	0.089	0.073	0.484	0.571
SLEDAI-2K at week 4	**<0.001**	**0.025**	**<0.001**	**<0.001**
SLEDAI-2K at week 8	**<0.001**	0.060	**<0.001**	**<0.001**
SLEDAI-2K at week 12	**<0.001**	0.093	**<0.001**	**<0.001**
SLEDAI-2K at week 16	**<0.001**	**0.038**	**0.002**	**<0.001**
SLEDAI-2K at week 20	**<0.001**	**0.045**	**0.003**	**<0.001**
SLEDAI-2K at week 24	**<0.001**	0.094	**0.005**	**<0.001**
SLEDAI-2K at week 28	**<0.001**	0.054	**0.004**	**<0.001**
SLEDAI-2K at week 32	**<0.001**	**0.008**	**0.002**	**<0.001**
SLEDAI-2K at week 36	**<0.001**	**0.008**	**0.001**	**<0.001**
SLEDAI-2K at week 40	**<0.001**	**0.042**	**<0.001**	**<0.001**
SLEDAI-2K at week 44	**<0.001**	0.097	**<0.001**	**<0.001**
SLEDAI-2K at week 48	**<0.001**	0.072	**<0.001**	**<0.001**
SLEDAI-2K at week 52	**<0.001**	0.256	**<0.001**	**<0.001**

Comparisons were performed using the one-way analysis of variance (ANOVA) test. Statistically significant *p* values are in bold.
SLEDAI-2K: SLE disease activity index 2000.

Supplementary Table S19. Across-cluster and pairwise comparisons regarding mean prednisone equivalent dose use from baseline through week 52.

	Overall comparison	Pairwise cluster 1 versus cluster 2	Pairwise cluster 1 versus cluster 3	Pairwise cluster 2 versus cluster 3
Prednisone equivalent dose				
Prednisone equivalent dose at baseline	**<0.001**	**<0.001**	0.330	**0.008**
Prednisone equivalent dose at week 4	**<0.001**	**<0.001**	0.782	**<0.001**
Prednisone equivalent dose at week 8	**<0.001**	0.095	0.423	**<0.001**
Prednisone equivalent dose at week 12	**<0.001**	0.087	0.252	**<0.001**
Prednisone equivalent dose at week 16	**0.001**	0.174	0.281	**<0.001**
Prednisone equivalent dose at week 20	**<0.001**	0.121	0.120	**<0.001**
Prednisone equivalent dose at week 24	**<0.001**	0.079	0.322	**<0.001**
Prednisone equivalent dose at week 28	**<0.001**	0.135	0.272	**<0.001**
Prednisone equivalent dose at week 32	**<0.001**	0.094	0.303	**<0.001**
Prednisone equivalent dose at week 36	**<0.001**	0.099	0.223	**<0.001**
Prednisone equivalent dose at week 40	**<0.001**	0.094	0.253	**<0.001**
Prednisone equivalent dose at week 44	**<0.001**	0.137	0.219	**<0.001**
Prednisone equivalent dose at week 48	**<0.001**	0.137	0.228	**<0.001**
Prednisone equivalent dose at week 52	**<0.001**	0.132	0.237	**<0.001**

Comparisons were performed using the one-way analysis of variance (ANOVA) test. Statistically significant *p* values are in bold.
