# Supplementary figures and images for: Unsupervised machine learning identifies distinct SLE patient endotypes with differential response to belimumab
Source: Rheumatology (Oxford). 2025 Apr 17;64(8):4650–8. doi: 10.1093/rheumatology/keaf215 (PMC12316369; doi:10.1093/rheumatology/keaf215)

Cluster Differences Between Train and Test Data

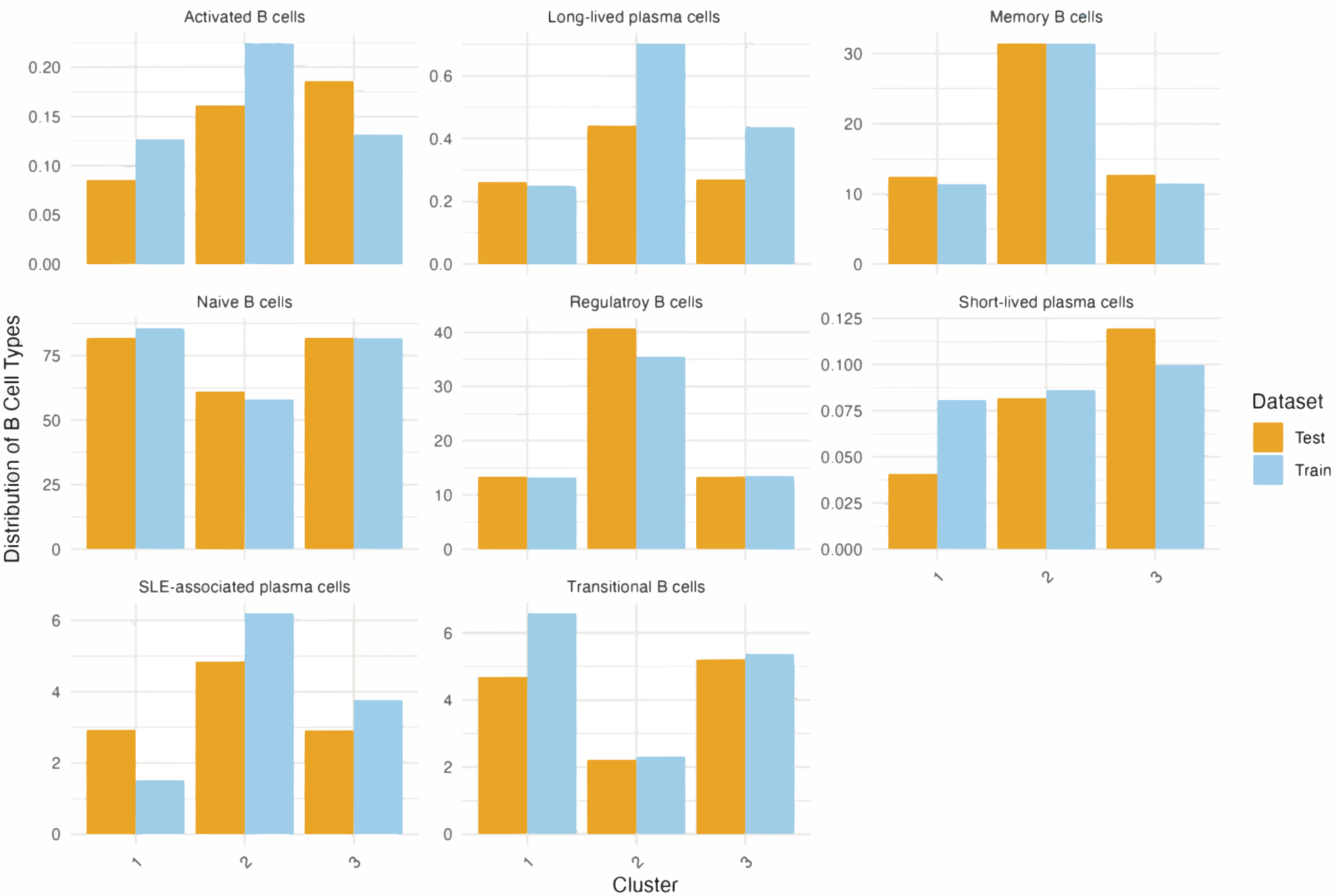

Supplement: keaf215_Supplementary_Data [file keaf215_supplementary_data.zip › rhe-24-2878-File008.pdf]
